# Exon junction complex-associated multi-adapter RNPS1 nucleates splicing regulatory complexes to maintain transcriptome surveillance

**DOI:** 10.1093/nar/gkac428

**Published:** 2022-05-30

**Authors:** Lena P Schlautmann, Jan-Wilm Lackmann, Janine Altmüller, Christoph Dieterich, Volker Boehm, Niels H Gehring

**Affiliations:** Institute for Genetics, University of Cologne, 50674 Cologne, Germany; Center for Molecular Medicine Cologne (CMMC), University of Cologne, 50937 Cologne, Germany; CECAD Research Center, University of Cologne, Joseph-Stelzmann-Str. 26, 50931 Cologne, Germany; Cologne Center for Genomics (CCG), University of Cologne, 50931 Cologne, Germany; Section of Bioinformatics and Systems Cardiology, Department of Internal Medicine III and Klaus Tschira Institute for Integrative Computational Cardiology, Heidelberg University Hospital, 69120 Heidelberg, Germany; DZHK (German Centre for Cardiovascular Research), Partner site Heidelberg/Mannheim, 69120 Heidelberg, Germany; Institute for Genetics, University of Cologne, 50674 Cologne, Germany; Center for Molecular Medicine Cologne (CMMC), University of Cologne, 50937 Cologne, Germany; Institute for Genetics, University of Cologne, 50674 Cologne, Germany; Center for Molecular Medicine Cologne (CMMC), University of Cologne, 50937 Cologne, Germany

## Abstract

The exon junction complex (EJC) is an RNA-binding multi-protein complex with critical functions in post-transcriptional gene regulation. It is deposited on the mRNA during splicing and regulates diverse processes including pre-mRNA splicing and nonsense-mediated mRNA decay (NMD) via various interacting proteins. The peripheral EJC-binding protein RNPS1 was reported to serve two insufficiently characterized functions: suppressing mis-splicing of cryptic splice sites and activating NMD in the cytoplasm. The analysis of transcriptome-wide effects of EJC and RNPS1 knockdowns in different human cell lines supports the conclusion that RNPS1 can moderately influence NMD activity, but is not a globally essential NMD factor. However, numerous aberrant splicing events strongly suggest that the main function of RNPS1 is splicing regulation. Rescue analyses revealed that the RRM and C-terminal domain of RNPS1 both contribute partially to regulate RNPS1-dependent splicing events. We defined the RNPS1 core interactome using complementary immunoprecipitations and proximity labeling, which identified interactions with splicing-regulatory factors that are dependent on the C-terminus or the RRM domain of RNPS1. Thus, RNPS1 emerges as a multifunctional splicing regulator that promotes correct and efficient splicing of different vulnerable splicing events via the formation of diverse splicing-promoting complexes.

## INTRODUCTION

The majority of human genes contain introns and their transcribed pre-mRNAs are subject to (alternative) splicing (1). During splicing, intronic sequences are excised and exons are ligated by the spliceosome, resulting in a mature mRNA that is subsequently exported to the cytoplasm (2). The spliceosome has the critical as well as delicate task to identify the correct splice sites, because frequently there is more than one possible splice site. These additional sites can be designated alternative splice sites, but also so-called cryptic splice sites (3). While the usage of the former can be employed to generate different transcript isoforms, the erroneous utilization of cryptic splice sites leads to mis-splicing and the production of defective transcripts (4). Both processes use similar mechanisms, but with opposing results. Alternative splicing (AS) increases the number of protein isoforms produced from a single gene by the varying usage of 5′ and 3′ splice sites, skipping of exons and inclusion of introns (5). In addition, it is also an important step in gene expression regulation. In contrast, the use of cryptic splice sites is often associated with the production of non-functional transcripts and the occurrence of disease (4). Therefore, (alternative) splicing requires tight regulation and accurate operation of the spliceosome to ensure the production of the correct mature mRNAs.

The recognition of the proper splice sites by the spliceosome is assisted by auxiliary proteins, which bind to the pre-mRNA and guide the spliceosome to the correct positions (6). The group of splicing regulatory proteins is quite diverse and includes several RNA-binding proteins that interact with specific sequence motifs, such as the SR proteins (7). The class of SR proteins is characterized by one or two N-terminal RNA binding domains (e.g. RRMs), and a C-terminal domain enriched in arginine and serine dipeptides (RS-domain). SR proteins commonly bind to exonic splicing enhancers (ESEs) and thereby define the exons to be maintained in the mature mRNA (8). In contrast, heterogeneous nuclear ribonucleoproteins (hnRNPs) bind mainly to intronic sequences (intronic splicing enhancers, ISEs) and support their recognition and removal by the spliceosome (9,10). The loss or exchange of ESE and ISE sequences can have dramatic effects on the splicing pattern and might even result in the inactivation of genes (11). However, the splicing process is not only regulated by SR proteins and hnRNPs, but also by many other RNA-binding proteins (12). Among these is the exon junction complex (EJC), an RNA-binding protein complex, which binds 24 nt upstream of an emerging exon-exon junction, independent of the RNA sequence (13). The EJC core consists of the three proteins EIF4A3, RBM8A and one of the MAGOH paralogs (MAGOH or MAGOHB, (14)) that are deposited onto the mRNA during splicing by interactions of the EJC proteins with spliceosome components (15,16).

The EJC carries out diverse functions during post-transcriptional gene regulation and besides regulating pre-mRNA splicing, it also facilitates the transport and translation of spliced mRNAs (13). Furthermore, the EJC is critical for nonsense-mediated mRNA decay (NMD) and EJC proteins were initially described to enable the detection of NMD substrates, particularly mRNAs containing premature translation termination codons (PTCs) (17,18). NMD not only serves as a quality control mechanism by ensuring the degradation of incorrect mRNAs, but is also important for the regulation of gene expression (19). NMD substrates can be produced in different ways: NMD-activating termination codons may result from AS or genomic mutations, in other cases NMD is triggered by a long 3′ UTR or usage of an upstream open reading frame (uORF). Efficient NMD takes place when a ribosome terminates at a PTC. EJCs bound to the mRNA downstream of that PTC will serve as a signal for the NMD machinery to initiate the degradation of that mRNA. Therefore, the EJC is essential for NMD to correctly identify transcripts that need to be degraded.

In order to carry out all its different tasks, the EJC functions as a binding platform for auxiliary factors which itself have varying regulatory potentials. Two of the EJC-associated complexes, the ACIN1-containing apoptosis and splicing associated protein complex (ASAP) and the related PNN-containing PSAP complex are known regulators of splicing (20). ASAP and PSAP complexes share two of their components, RNA binding protein with serine rich domain 1 (RNPS1) and Sin3A associated protein 18 (SAP18), but vary in the third component, which is either Acinus (ACIN1) or Pinin (PNN), respectively (21,22). A previous study has shown that knockdown (KD) of PNN and ACIN1 affects different splicing events, suggesting that ASAP and PSAP complexes have non-overlapping functions in splicing regulation (23). In *D. melanogaster*, retention of PIWI intron 4 relies on the EJC core, ACIN1 and RNPS1 (24,25). Recent studies furthermore demonstrate the ability of the EJC core and the PSAP to suppress the usage of cryptic 5′ and 3′ splice sites (26–29). While cryptic 3′ splice sites are suppressed by direct masking by the EJC core, the suppression of cryptic 5′ splice sites involves an unknown mechanism requiring RNPS1 recruitment via the EJC core and the PSAP complex (26,27). It is likely that RNPS1 represents the central functional component in all these processes, whereas ACIN1 as well as probably PNN play a role in RNPS1 recruitment. ACIN1, for example, directly binds to the EJC core, which would explain how the interaction of RNPS1 and the EJC is established.

In addition to its function in splicing, RNPS1 has the ability to activate NMD when tethered to a reporter mRNA downstream of the termination codon (18,30). It has also been reported that the presence of RNPS1 on NMD-targeted mRNAs leads to more pronounced degradation (31). However, there are controversial results as to whether RNPS1 has an essential role in NMD or not (32,33). Although the exact function of RNPS1 during NMD remains to be determined, it is clear that RNPS1 interacts with the EJC and possibly also with components of the NMD machinery, potentially forming a bridge between these two macromolecular assemblies.

Although previous work had examined individual aspects of RNPS1, its function in the context of the EJC is still not fully understood and therefore demands a more comprehensive characterization of RNPS1. In this study, we uncover that RNPS1 only mildly affects a small subset of NMD targets, whereas its main function is the regulation of alternative splicing and the suppression of cryptic splice sites. Several domains of RNPS1 engage in splicing regulation. The RNPS1 RRM, which is known to be required for ASAP/PSAP assembly, regulates splicing by recruiting other splicing factors, including spliceosomal components (21). We also identified components of the U1 snRNP, members of the LUC7L family and other splicing factors that interact with the C-terminus. Thus, we conclude that the individual domains of RNPS1 contribute to bridge different splicing competent complexes to the EJC, thereby regulating splicing of surrounding/adjacent introns. In our model RNPS1 acts as a multi-functional adapter that recruits splicing factors independently of the mRNA sequence to the EJC binding site.

## MATERIALS AND METHODS

### Cell culture

Flp-In-T-REx-293 (HEK293 or 293; human, female, embryonic kidney, epithelial; Thermo Fisher Scientific, RRID:CVCL_U427), HeLa Flp-In-T-REx (HeLa FT; human, female, cervix; Elena Dobrikova and Matthias Gromeier, Duke University Medical Center) and HeLa Tet-Off (HTO; human, female, cervix; Clontech, RRID:CVCL_V352) cells were cultured in high-glucose, GlutaMAX DMEM (Gibco) supplemented with 9% fetal bovine serum (Gibco) and 1x Penicillin Streptomycin (Gibco). The cells were cultivated at 37°C and 5% CO_2_ in a humidified incubator. The generation of stable cell lines is described below and all cell lines are summarized in Supplementary Table S1.

### Stable cell lines and plasmids

RNPS1 point and deletion mutants were PCR amplified using Q5 polymerase (New England Biolabs) and inserted into PB-CuO-MCS-BGH-EF1-CymR-Puro (modified from System Biosciences), together with an N-terminal FLAG-emGFP-tag, FLAG-TurboID-tag or MYC-UltraID-tag via NheI and NotI (both New England Biolabs) restriction sites. As a control, FLAG-emGFP, FLAG-TurboID-NLS or MYC-UltraID-NLS was equally cloned into the PB-CuO-MCS-BGH-EF1-CymR-Puro vector.

HEK293 and HTO cells were stably transfected using the PiggyBac Transposon system. 2.5–3 × 10^5^ cells were seeded 24 h before transfection in 6-wells. 1 μg of PiggyBac construct was transfected together with 0.8 μg of the Super PiggyBac Transposase expressing vector using the calcium phosphate method. 48 h after transfection, the cells were transferred into 10 cm dishes and selected with 2 μg ml^–1^ puromycin (InvivoGen). After 7–10 days, the colonies were pooled. Expression of the PiggyBac constructs was induced with 30 μg ml^–1^ cumate.

RFX5 reporters were PCR amplified as described above and cloned into pcDNA5/FRT/TO/FLAG. HeLa FT cells were stably transfected with the reporters using the Flp-In-T-REx system. Transfection and selection were performed like for PiggyBac transfected cells, with the following differences: 1.5 μg of pcDNA5 construct were co-transfected with 1.5 μg Flippase expression vector (pOG44) and cells were selected with 100 μg ml^–1^ hygromycin (InvivoGen). All cell lines generated and plasmids used in this study are listed in Supplementary Table S1.

### Co-immunoprecipitation

Expression of FLAG-emGFP tagged RNPS1 mutants and FLAG-emGFP control was induced in stable cell lines (1.5 × 10^6^ cells per 10 cm dish) using cumate (as described above) 72 h before cell lysis. The samples were lysed in 600 μl buffer E (20 mM HEPES–KOH (pH 7.9), 100 mM KCl, 10% glycerol, 1 mM DTT, Protease Inhibitor) in the presence of 1 μg ml^–1^ RNase A and sonicated using the Bandelin Sonopuls mini20 with 10 pulses (2.5 mm tip, 1s pulse, 50% amplitude). For immunoprecipitation, the protein concentration of the lysates was measured using Pierce Detergent Compatible Bradford Assay Reagent (Thermo Fisher Scientific) and adjusted in buffer E. Then, the lysates were loaded onto Anti-FLAG M2 Magnetic Beads (Sigma-Aldrich) and incubated for 2 h at 4°C with overhead shaking. After that, the beads were washed four times for 3 min with mild wash buffer (20 mM HEPES-KOH (pH 7.9), 137 mM NaCl, 2 mM MgCl_2_, 0.2% Triton X-100, 0.1% NP-40). For elution, 2 × 21.5 μl (42.5 μl total) of a 200 mg ml^–1^ dilution of FLAG peptides (Sigma) in 1× TBS was used.

### TurboID/UltraID proximity labeling

The proximity labeling was performed in a similar way as described before by (34). Here, stable cell lines were used for inducible expression of FLAG-TurboID or MYC-UltraID-tagged RNPS1 constructs (Supplementary Table S1) (35–37). These cell lines were seeded at a density of 1.5 × 10^6^ cells per 10 cm dish and after 72 h, medium was exchanged to medium containing dialyzed FBS instead of non-dialyzed FBS (Gibco; A3382001; to suppress background biotinylation)(38) and cumate to induce expression of TurboID/UltraID-tagged RNPS1 (as described above).

On the next day, 50 μM biotin was added for 10 minutes followed by two washing steps with PBS and scraping of the cells in 1 ml ice cold PBS. Samples were centrifuged for 5 min at 100 × g and 4°C, and resuspended in 200 μl phospho-RIPA buffer (50 mM Tris pH 8.0, 150 mM NaCl, 1% IGEPAL CA 630, 0.5% deoxycholate, 0.1% SDS, 1 μg/ml RNase A) supplemented with 1 tablet of PhosSTOP (Roche), 100 μl EDTA-free HALT Protease & Phosphatase Inhibitor (ThermoFisher) per 10 ml buffer. Samples were sonicated and protein concentrations were measured as described above for co-immunoprecipitation. 50 μl input aliquots containing 100 μg of total protein were prepared and mixed with SDS-sample buffer. 1 mg protein in 500 μl buffer were loaded onto 0.5 ml Amicon Ultra centrifugal filter devices (3K cutoff) which were centrifuged for 45 min at 4°C and 14 000 × g, to concentrate samples to approximately 100 μl and minimize excess biotin in the sample. For enrichment of biotinylated proteins, samples were combined with 200 μl RIPA buffer (wash of centrifugal filter), added to 25 μl pre-washed Pierce Streptavidin Magnetic Beads (ThermoFisher) and incubated for 2 h at 4°C with overhead shaking. Four washing steps with 800 μl RIPA buffer for 5 min were followed by one wash with 800 μl mild wash buffer (20 mM HEPES–KOH (pH 7.9), 137 mM NaCl, 2 mM MgCl_2_, 0.2% Triton X-100, 0.1% NP-40). For elution of biotinylated proteins, the beads were incubated for 15 min @ RT with 50 μl 5% SDS, supplemented with 20 mM DTT and 3 mM biotin, then for 15 min at 96°C, followed by another elution with 25 μl at both temperatures. Lastly, the resulting two eluates were combined and incubated for 30 minutes at 55°C followed by addition of 8.5 μl of 400 mM CAA (final concentration of 40 mM) to alkylate the eluates and 30 min incubation in the dark. Tryptic protein digestion and proteomics analysis was performed as described below.

### Label-free mass spectrometry and computational analysis

For label-free mass spectrometry, immunoprecipitated samples were prepared as described above and after addition of 1 volume of 5% SDS in PBS reduced with DTT and alkylated with CAA (final concentrations 5 and 55 mM, respectively). Proximity-labeled samples were prepared and alkylated as described in the prior section. The following steps were performed for both sample types (immunoprecipitated and proximity-labeled samples). For tryptic protein digestion, a modified version of the single pot solid phase-enhanced sample preparation (SP3) protocol was used as described below (39). Afterwards, proteins were supplemented with paramagnetic Sera-Mag speed beads (Cytiva) and mixed in a 1:1-ratio with 100% acetonitrile (ACN). After 8 min incubation, protein-beads-complexes were captured using an in-house build magnetic rack, washed twice with 70% EtOH, and washed once with 100% ACN. After air-drying and reconstitution in 5 μl 50 mM triethylammonium bicarbonate, samples were supplemented with 0.5 μg trypsin and 0.5 μg LysC and incubated overnight at 37°C. The beads were resuspended on the next day and mixed with 200 μl ACN, followed by 8 min incubation. Subsequently, the samples were placed on the magnetic rack to wash the tryptic peptides once with 100% ACN. Samples were air-dried, dissolved in 4% DMSO, transferred into new PCR tubes, and acidified with 1 μl of 10% formic acid. Proteomics analysis was performed by the proteomics core facility at CECAD via data-dependent acquisition using an Easy nLC1200 ultra high-performance liquid chromatography (UHPLC) system connected via nano electrospray ionization to a Q Exactive Plus instrument (all Thermo Scientific) running in DDA Top10 mode. Based on their hydrophobicity the tryptic peptides were separated using a chromatographic gradient of 60 min with a binary system of buffer A (0.1% formic acid) and buffer B (80% ACN, 0.1% formic acid) with a total flow of 250 nl/min. Separation was achieved on in-house made analytical columns (length: 50 cm, inner diameter: 75 μm) containing 2.7 μm C18 Poroshell EC120 beads (Agilent) heated to 50°C in a column oven (Sonation). Over a time period of 41 min, Buffer B was linearly increased from 3% to 30% followed by an increase to 50% in 8 min. Finally, buffer B was increased to 95% within 1 min followed by 10 min washing step at 95% B. Full mass spectrometry (MS) spectra (300–1,750 *m*/*z*) were recorded with a resolution of 70,000, a maximum injection time of 20 ms and an AGC target of 3e6. In each full MS spectrum, the top 10 most abundant ions were selected for HCD fragmentation (NCE 27) with a quadrupole isolation width of 1.8 m/z and 10 s dynamic exclusion. The MS/MS spectra were then measured with a 35,000 resolution, an injection time of maximum 110 ms and an AGC target of 5e5.

The MS RAW files were then analyzed with MaxQuant suite (version 1.5.3.8) on standard settings. By matching against the human UniProt database the peptides were then identified using the Andromeda scoring algorithm (40). Carbamidomethylation of cysteine was defined as a fixed modification, while methionine oxidation and N-terminal acetylation were variable modifications. The digestion protein was Trypsin/P. A false discovery rate (FDR) <0.01 was used to identify peptide-spectrum matches and to quantify the proteins. Data processing and statistical analysis were performed in the Perseus software (version 1.6.15.0) (41). For further analysis, gene ontology biological process (GOBP) terms were obtained from Uniprot (42) using the majority Protein ID and subsequently parsed for the terms ‘splic’, ‘RNA processing’, ‘ribonucleoprotein|RNA binding’, ‘mRNA’ to define relevant GOBPs.

### Immunoblot analysis

Protein samples from co-immunoprecipitation were loaded onto SDS-polyacrylamide gels using SDS-sample buffer, separated by gel-electrophoresis and analysed by immunoblotting. All antibodies were diluted in 50 mM Tris [pH 7.2], 150 mM NaCl with 0.2% Tween-20 and 5% skim milk powder. Antibodies and dilutions are listed in Supplementary Table S1. For visualization, we used Amersham ECL Prime or Select Western Blotting Detection Reagent (GE Healthcare) in combination with the Fusion FX-6 Edge system (Vilber Lourmat). Quantification was performed in a semi-automated manner using the ImageQuant TL 1D software (GE Healthcare Life Sciences) with a rolling-ball background correction.

### Protein structure modelling and visualization

Chimera X Version 1.1 was used to visualize the structure of the ASAP complex (accession number 4A8X on PDB, (21)).

### siRNA-mediated knockdowns

2–3 × 10^5^ cells were seeded in 6-well plates well and reverse transfected using 2.5 μl Lipofectamine RNAiMAX and a total of 60 pmol of the respective siRNA(s) according to the manufacturer's instructions. All siRNAs used in this study are listed in Supplementary Table S1.

### RNA extraction, reverse transcription, endpoint and quantitative RT-PCR

RNA was extracted using different extraction methods. For endpoint or quantitative RT-PCR (RT-qPCR), RNA was extracted using peqGOLD TriFast (VWR Peqlab) or RNA-Solv Reagent (Omega Bio-Tek) following the manufacturer's instructions for TriFast but using 150 μl 1-bromo-3-chloropropane instead of 200 μl chloroform and eluting the RNA in 20 μl RNase-free water. Reverse Transcription was performed using the GoScript Reverse Transcriptase (Promega), 10 μM VNN-(dT)_20_ primer and 0.5–1 μg of total RNA in a 20 μl reaction volume. RT-PCR and RT-qPCR were performed according to the manufacturer's protocols using MyTaq™ Red Mix (Bioline/BIOCAT) for RT-PCR and GoTaq qPCR Master Mix (Promega) for RT-qPCR. For quantification of RT-PCRs, the Image Lab 6.0.1 software (Bio-Rad) was used. All primers used in this study are listed in Supplementary Table S1.

### RNA-Sequencing and computational analysis

HTO or HEK293 cells and the indicated rescue cell lines were treated with siRNA as described above. HTO sets were harvested using TriFast, the HEK293 set was harvested using RNA Solv Reagent. Extraction of total RNA was performed with DIRECTzol Miniprep Kit (Zymo Research), according to the manufacturer's instructions.

For each sample, three biological replicates were analyzed. The Spike-In Control Mix (SIRV Set1 SKU: 025.03, Lexogen), which enables performance assessment by providing a set of external RNA controls, was added to the total RNA, as listed in Supplementary Table S1. The Spike-Ins were used for quality control purposes, but not used for the final analysis of differential gene expression (DGE), differential transcript usage (DTU) or alternative splicing (AS). The cDNA library was prepared using the TruSeq Stranded Total RNA kit (Illumina). Library preparation involved the removal of ribosomal RNA using biotinylated target-specific oligos combined with Ribo-Zero Gold rRNA removal beads from 1 μg total RNA input. the Ribo-Zero Human/Mouse/Rat kit depleted cytoplasmic and mitochondrial rRNA from the samples. Following a purification step, the remaining RNA was fragmented and cleaved. The first strand cDNA was synthesized using reverse transcriptase and random primers. Subsequently, the second strand cDNA synthesis was performed using DNA Polymerase I and RNase H. To the resulting double-stranded cDNA, a single ‘A’ base was added and the adapters were ligated. After this, the cDNA was purified and amplified with PCR, followed by library validation and quantification on the TapeStation (Agilent). Equimolar amounts of library were pooled and quantified using the Peqlab KAPA Library Quantification Kit 587 and the Applied Biosystems 7900HT Sequence Detection System. Sequencing was performed on an Illumina NovaSeq6000 sequencing instrument with an PE100 protocol.

The resulting reads were aligned against the human genome (version 38, GENCODE release 33 transcript annotations (43), supplemented with SIRVomeERCCome annotations from Lexogen; (obtained from https://www.lexogen.com/sirvs/download/) using the STAR read aligner (version 2.7.3a) (44). Salmon (version 1.3.0) (45) was used to compute estimates for transcript abundance with a decoy-aware transcriptome. After the import of transcript abundances using tximport (46) and filtering for genes with at least 10 counts in all samples, differential gene expression analysis was performed with the DESeq2 (47) R package (version 1.34.0) with the significance thresholds |log2FoldChange| > 1 and adjusted *P*-value (*P*adj) < 0.05. Differential splicing was detected with LeafCutter (version 0.2.9) (48) with the significance thresholds |deltaPSI| > 0.1 and *P*adj < 0.001. Alternatively, rMATS (version 4.1.1, (49)) with novel splice site detection was used to identify alternative splicing (AS) classes, followed by analysis using maser (version 1.8.0) and significance thresholds |deltaPSI| > 0.2 and *P*adj < 0.01.

Differential transcript usage was computed with IsoformSwitchAnalyzeR (ISAR, version 1.16.0) and the DEXSeq method (46,50–54). Significance thresholds were delta isoform fraction |dIF| > 0.1 and adjusted *P*-value (isoform_switch_q_value) < 0.001. PTC status of transcript isoforms with annotated open reading frame was determined by IsoformSwitchAnalyzeR using the 50 nucleotide (nt) rule of NMD (51,55–57). Intron retention was computed with IRFinder (version 1.2.6, (58)) in FastQ mode and differential intron retention was calculated using DESeq2 with the significance thresholds |log_2_FoldChange| > 1 and *P*adj < 0.001. Sashimi plots were generated using ggsashimi (version 1.0.0, (59)). All cutoffs stated above were set as defaults, but may differ on individual plots as indicated.

### Data presentation

Quantifications and calculations for other experiments were performed—if not indicated otherwise—with Microsoft Excel (version 1808) or R (version 4.1.2) and all plots were generated using either IGV (version 2.8.12), GraphPad Prism 5, ggplot2 (version 3.3.5) or ComplexHeatmap (version 2.10.0) (60). Overlaps of data sets were represented via nVennR (version 0.2.3) (61) or the ComplexHeatmap package (version 2.10.0) (60).

## RESULTS

### RNPS1 expression levels regulate a subset of NMD targets

**Figure 1.**
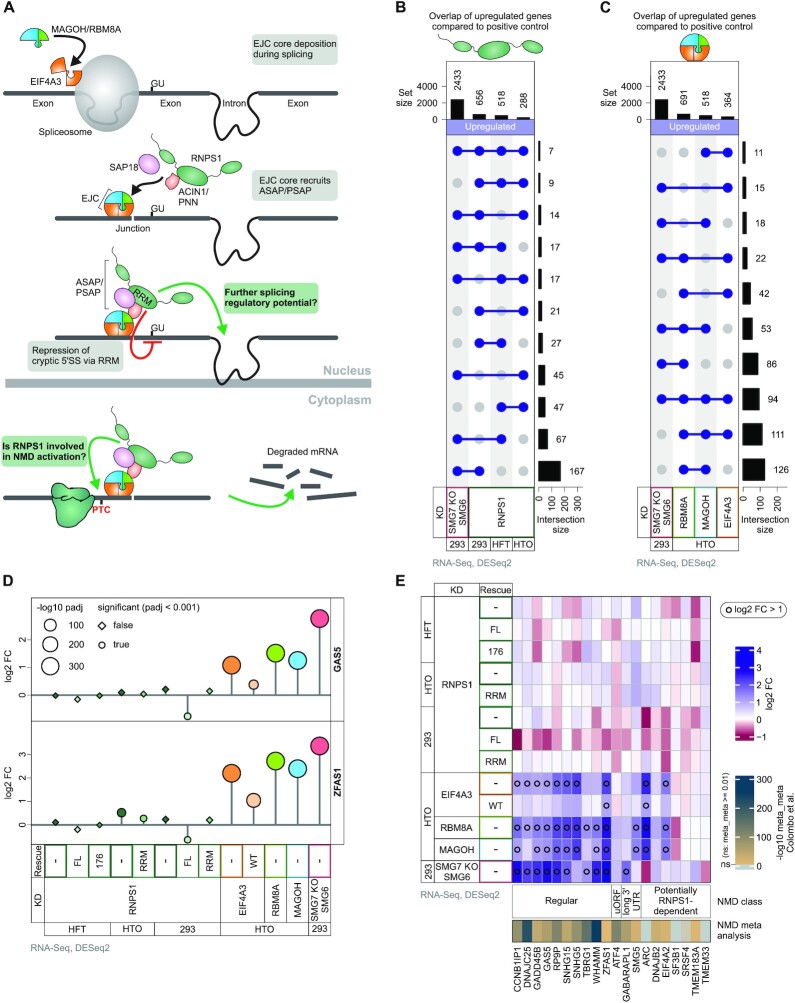
RNPS1 expression levels mildly influence selected NMD events. (**A**) Schematic depiction of exon junction complex (EJC) deposition on mRNAs during splicing, recruitment of RNPS1-containing ASAP or PSAP complexes to EJCs, alternative splicing (AS) regulation (including cryptic 5′ splice site suppression by RNPS1 RRM domain) and nonsense-mediated mRNA decay (NMD) activation by RNPS1. Grey boxes indicate established functions and green boxes indicate uncertain functions of RNPS1, which are investigated in this manuscript. (B, C) Differential gene expression (DGE) was analyzed using DESeq2 and upregulated genes were identified (Cutoffs: log2 fold change (log2 FC) > 1 and adjusted *P*-value (*P*adj) < 0.05). The intersections between the selected RNA-Seq conditions are depicted in UpSet plots for (**B**) RNPS1 KDs in the different cell types compared to SMG7 KO + SMG6 KD in HEK293 cells and (**C**) EJC KDs in HTO cells compared to SMG7 KO + SMG6 KD in HEK293 cells. *P*-values were calculated by DESeq2 using a two-sided Wald test and corrected for multiple testing using the Benjamini-Hochberg method. (**D**) DGE analysis of GAS5 and ZFAS1 log_2_ fold change (log2 FC) in the indicated RNA-Seq conditions as compared to the corresponding control. Circle/square size depicts the −log_10_ adjusted *P*-value (*P*adj), the shape depicts whether the expression change is significant (true) or non-significant (false) (cutoff: *P*adj < 0.001). (**E**) Heatmap of log_2_ fold change (log_2_ FC) of selected previously identified NMD target genes. Black circles indicate log_2_ FC above 1. Values of an NMD meta-analysis (66) are indicated.

RNPS1 was shown to regulate multiple types of AS in combination with other ASAP/PSAP components in *D. melanogaster* and human cells (23–26). Furthermore, several studies indicated that RNPS1 is able to activate NMD (30,31,62,63) and more recently RNPS1 was reported to be involved in the recognition of many EJC-dependent NMD substrates (32) (Figure 1A). To investigate the role of RNPS1 in NMD in the context of the EJC, we performed RNA-sequencing (RNA-Seq) analyses of cultured human cells depleted of either RNPS1 or the EJC core factors EIF4A3, MAGOH or RBM8A (Supplementary Figure S1A). Additionally, we sequenced RNA from stable cell lines (over)expressing siRNA-insensitive RNPS1 (FLAG-emGFP-tagged) or EIF4A3 constructs to rescue the respective siRNA mediated knockdown (KD) (Supplementary Figure S1A-C). In total, we generated three new RNA-Seq datasets from Flp-In-T-REx-293 (HEK293 or 293) cells and HeLa Tet-Off (HTO) cells and re-analyzed existing RNA-Seq datasets of RNPS1 KD-rescue in HeLa Flp-In-T-REx (HFT; E-MTAB-6564) (26).

Since NMD normally destabilizes target mRNAs, we expected that transcriptome-wide impairment of NMD by depleting RNPS1 should lead to a marked upregulation of genes. However, global differential gene expression (DGE) analysis using DESeq2 identified almost as many or even more downregulated (497–518) than upregulated (288–656, depending on the cell line used) genes in the different RNPS1 KDs (Supplementary Figure S1D, Table S2). Furthermore, only 16 genes were consistently up- and 32 genes downregulated in all three RNPS1 KD cell lines, indicating that many DGE events were cell-type specific or stochastic variations (Supplementary Figure S1D). As expected for RNPS1-dependent effects, the complementation of the KD with full-length RNPS1 (RNPS1 FL) constructs in HEK293 and HFT cells substantially attenuated the differential gene expression (Supplementary Figure S1E). Of note, a rescue construct consisting of the RNPS1 RRM domain was considerably less able to restore normal expression of the mis-regulated genes. Similarly, the expression of an RNPS1 mutant unable to interact with the ASAP/PSAP complex and the EJC (RNPS1 176) (26) largely failed to rescue the RNPS1-dependent differentially expressed genes (Supplementary Figure S1E).

We hypothesized that if RNPS1 is indeed required for NMD, many NMD-targeted genes should be upregulated upon RNPS1 KD. As a reference for NMD-targeted genes, we used the DGE analysis of a recent RNA-Seq dataset from SMG7 knockout (KO) HEK293 cells with additional SMG6 KD ((34); E-MTAB-9330), which displayed nearly complete NMD inhibition, resulting in 2433 upregulated genes. Of the 656 upregulated genes in RNPS1 depleted HEK293 cells, in total 205 were also found in the NMD inhibited SMG7 KO + SMG6 KD data (Figure 1B). When we likewise compared EJC KDs, we found a total of 255 overlapping genes between the SMG7 KO + SMG6 KD and the RBM8A KD, which had a slightly higher number of upregulated genes than the RNPS1 KD (Figure 1C). Although the total amount of genes overlapping with SMG7 KO + SMG6 KD was similar for RBM8A and RNPS1, only seven genes were consistently upregulated in all three RNPS1 knockdowns and the SMG7 KO + SMG6 KD condition (Figure 1B). In contrast, the cumulative overlap between all three EJC-KDs and the SMG7 KO + SMG6 KD was higher and comprised 94 genes. This number is particularly remarkable considering that the EJC KDs targeted three different EJC proteins and were carried out in HTO and not HEK293 cells (Figure 1C). Furthermore, when visualizing the extent of differential gene expression of the known NMD targets ZFAS1 and GAS5 (snoRNA host genes) (64), we observed only mild effects (log_2_ fold change < 1) of RNPS1 KD compared to the stronger upregulation upon EJC or SMG6-SMG7 depletion (Figure 1D). We next investigated a broader selection of previously identified NMD targets, including genes harboring long 3′ UTR (SMG5, GABARAPL1; (65)) or upstream open reading frames (ATF4; (66)) as well as potential RNPS1-dependent targets (32) (Figure 1E). The DGE analysis of EJC or SMG6-SMG7 depleted conditions could not confirm the strong upregulation of all of these previously identified NMD targets (e.g. SF3B1, SRSF4 and TMEM33), which was supported by non-significant scores in a recent NMD meta-analysis (66). Importantly, none of the RNPS1 KD or rescue conditions altered the expression of any of the selected NMD targets above the effect strength cutoff (log_2_ fold change > 1; Figure 1D, E). However, the overexpression of RNPS1 FL in HEK293 cells frequently led to further downregulation of selected known NMD targets, suggesting that elevated RNPS1 levels can enhance NMD as previously reported (31).

**Figure 2.**
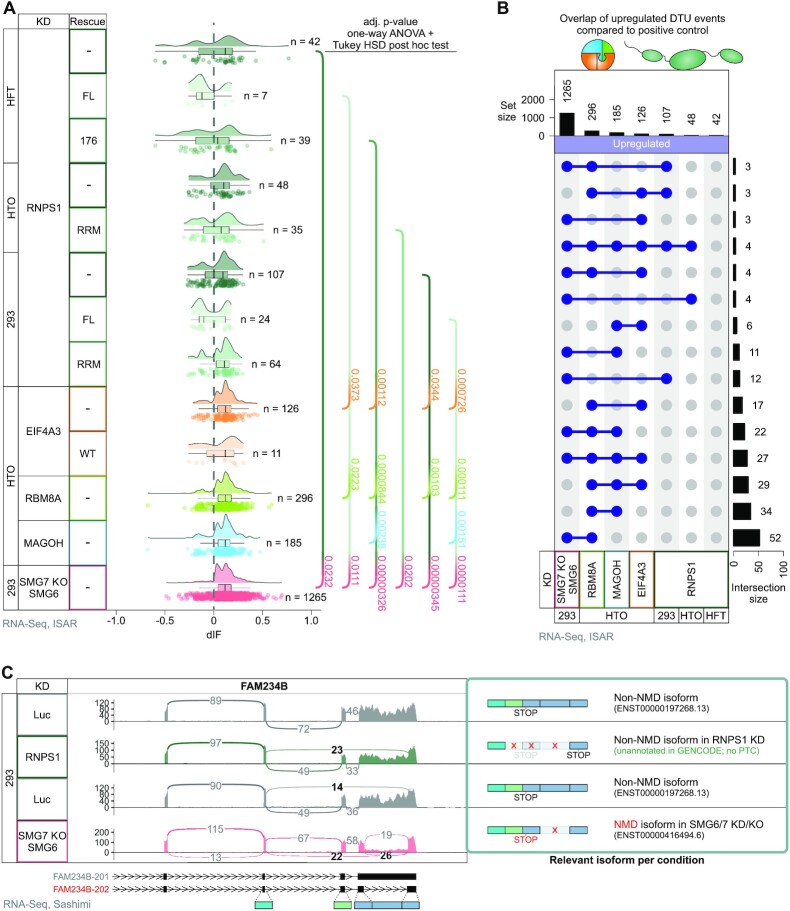
Differential transcript usage analysis reveals small overlap between RNPS1 knockdown and NMD-compromised conditions. (**A**) Raincloud plot depicting the change in isoform fraction (delta isoform fraction, dIF) for GENCODE (release 33) annotated premature translation termination codon (PTC)-containing isoforms (determined via IsoformSwitchAnalyzeR, ISAR) in the indicated RNA-Seq data. The number of individual events with FDR corrected *P*-value (isoform_switch_q_value) < 0.001 is indicated on the right. *P*-values were calculated by IsoformSwitchAnalyzeR using a DEXSeq-based test and corrected for multiple testing using the Benjamini-Hochberg method. The means of each condition were compared using a one-way ANOVA plus Tukey Honest Significant Differences (HSD) post hoc test and the adjusted *P*-value for significant comparisons are shown. (**B**) UpSet plot showing the top 15 intersections between the different RNA-Seq conditions with respect to differential transcript usage (DTU) (Cutoffs: FDR corrected *P*-value (isoform_switch_q_value) < 0.001). (**C**) Sashimi plot for FAM234B show the mean junction coverage of the indicated RNA-Seq data with the canonical and NMD-sensitive isoforms with their corresponding transcript ID depicted on the right. NMD-relevant and alternatively spliced junctions are highlighted and NMD isoforms are labeled in red.

NMD generally acts on the transcript-level and global DGE analyses fail to detect isoform-specific NMD events in which overall gene expression is largely unaltered. Therefore, we further characterized the role of RNPS1 in NMD by analyzing differential transcript usage (DTU) using the IsoformSwitchAnalyzeR package (ISAR) (52). This approach detects upregulated transcripts with annotated PTCs, which indicates NMD inhibition. Depletion of RNPS1 in all three cell lines (HEK293, HTO and HFT) caused a noticeable upregulation of transcripts bearing a PTC (Figure 2A, Supplementary Table S3), which was quantitatively less pronounced and statistically different than in SMG7 KO + SMG6 KD. Although this in principle supports a role of RNPS1 in the NMD process, we only found a minimal overlap of PTC-containing isoforms between RNPS1 KD and either EJC or SMG6-SMG7 depletion (Figure 2B). In contrast, the overlap between EJC core factor KDs and SMG6-SMG7 depletion was more robust. When plotted against each other, the event strength of differentially used transcripts found in both RBM8A KD and SMG7 KO + SMG6 KD showed good correlation (Supplementary Figure S2A). In contrast, there were considerably fewer shared transcripts between RNPS1 KD and SMG7 KO + SMG6 KD conditions, which also showed weaker correlation (Supplementary Figure S2B, C). To gain deeper insight into RNPS1’s NMD function, we visualized the RNA-Seq data for *bona fide* NMD targets, such as SRSF2, where the inclusion of an alternative exon and splicing of an intron in the 3′ untranslated region activates NMD. (67). Both NMD-activating AS events were clearly visible in the EJC- and SMG6-SMG7 depleted conditions, but comparably weak in the RNPS1 KDs (Supplementary Figure S2D).

Next, we focused on the overlapping PTC-containing transcripts that are upregulated both in the SMG7 KO + SMG6 KD NMD-compromised condition and in HEK293 RNPS1 KD. We manually inspected the expression profiles of these 16 designated PTC-containing mRNA isoforms and found that more than half cannot be classified as true NMD targets (Supplementary Figure S2E). One example is FAM234B, showing the highest delta isoform fraction (dIF) value in HEK293 RNPS1 KD, in which splicing of an intron in the 3′ untranslated region can produce an NMD isoform (Figure 2C). Accumulation of this PTC-containing isoform is detected in SMG6-SMG7 conditions, whereas in RNPS1- and EJC-depleted cells the skipping of two exons produces an isoform that is not predicted to trigger NMD (Figure 2C). This suggested that the ISAR algorithm may be mistaking unannotated isoforms in the RNPS1 KD for NMD isoforms. Indeed, we found three other examples of wrongly identified transcripts (Supplementary dataset S1). Three of the remaining twelve genes from the overlap show a downregulation of the NMD isoform upon RNPS1 KD compared to control KD, while for two other genes the total gene expression goes down while the expression of the NMD isoform remains unchanged. Seven of the overlapping transcripts can indeed be classified as true NMD isoforms in the RNPS1 KD condition, although their upregulation appears to be lower compared to SMG6-SMG7 depleted conditions (Supplementary Figure S2E, dataset S1).

In summary, the results from both DGE and DTU analyses suggest that RNPS1 can indeed influence NMD, but mostly in a mild and a rather transcript-specific than global manner. The expression levels of known NMD targets were moderately affected by RNPS1 depletion, whereas some targets, such as ZFAS1, were degraded more efficiently when RNPS1 FL was (over)expressed (Figure 1D, E). As previously reported (31), these observations indicate that the RNPS1 expression levels can influence NMD activity on selected targets. Considering the apparent cell type specific differences in DGE and DTU results, we wondered whether the RNPS1 expression levels differ in the three used cell lines. Quantification of normalized counts revealed that HEK293 cells expressed at least 2x more RNPS1 than the other cell lines (Supplementary Figure S2F). In combination with the better RNPS1 knockdown efficiency (log_2_ fold change −2.79), this result could explain the higher number of differentially used PTC-positive transcripts in this cell type. Nevertheless, the DTU in-depth analysis revealed that only about half of the potential high-confidence NMD targets (found in both RNPS1 KD as well as SMG6-SMG7 depletion) are indeed targeted by NMD and the remaining targets are mis-classified as a result of unannotated splicing or (NMD-insensitive) isoform up- or downregulation. Therefore, we conclude that RNPS1 acts mainly as an NMD activator on a small set of NMD substrates. However, the DTU analysis showed that RNPS1 is clearly involved in regulating different AS events (Figure 2C).

### Incomplete rescue of RNPS1 dependent alternative splicing events by RNPS1 RRM expression

**Figure 3.**
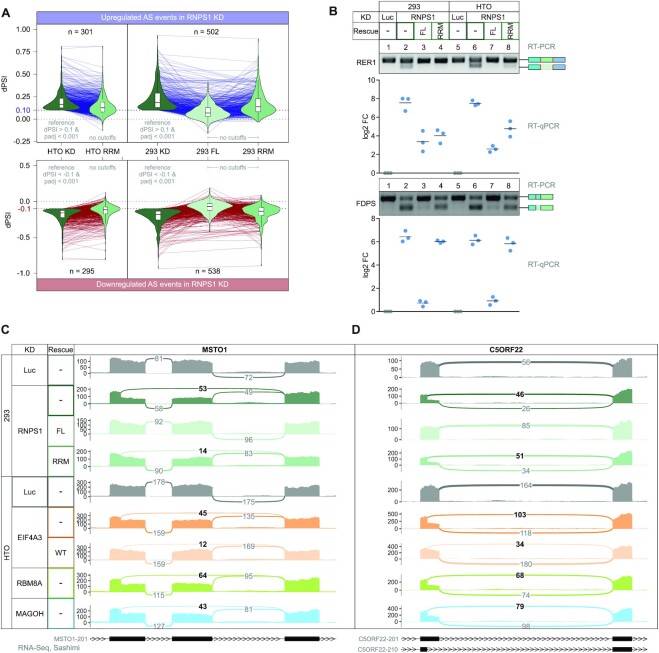
RNPS1 RRM domain partially rescues RNPS1-dependent alternative splicing events. (**A**) DeltaPSI (dPSI) values of AS events in the indicated RNA-Seq data were calculated with LeafCutter and depicted in a combined violin and parallel coordinate plot. Only alternative splicing events that are found in the RNPS1 KD conditions with the indicated cutoffs are plotted, no cutoffs were applied to the other conditions. *P*-values were calculated by LeafCutter using an asymptotic Chi-squared distribution and corrected for multiple testing using the Benjamini-Hochberg method. (**B**) Comparison of alternatively spliced transcript isoform abundance by RT-PCR and RT-qPCR of RER1 and FDPS in HEK293 or HTO KD and KD/rescue cells with the resulting PCR product indicated on the right. A representative replicate of the RT-PCRs (n = 3) is shown. The log_2_ fold change (log_2_ FC) of RT-qPCRs is calculated as the ratio of alternatively spliced to normally spliced transcript and plotted as datapoints and means. (**C**, **D**) Mean junction coverage in the indicated RNA-Seq conditions is depicted as a sashimi plots with the annotated transcript isoforms indicated below and the relevant alternative splice junctions highlighted. Event types are (**C**) Exon skipping (ES) and alternative 5′ splice site (A5SS) usage in MSTO1 and (**D**) A5SS usage in C5ORF22.

Encouraged by the findings of the DTU analysis, we next wanted to further characterize the function of RNPS1 in regulating AS. Previous studies detected hundreds of RNPS1-dependent AS events and experiments with a few individual transcripts demonstrated that the isolated RRM can rescue AS events caused by RNPS1 KD (26). To detect transcriptome-wide AS upon loss of RNPS1 or rescue with the RRM domain, we analyzed the RNA-Seq datasets with the intron-centric LeafCutter algorithm (48). Overall, we detected several hundred AS events in the RNPS1 KD conditions, with strong overlap between KD and RRM rescue conditions (Supplementary Figure S3A). In line with this observation, compared to the almost complete restoration of normal splicing by RNPS1 FL, about two-thirds of AS events could not be rescued by the RNPS1 RRM domain (Figure 3A, Supplementary Table S4). However, it proved difficult to define whether an AS event is fully rescued or not since the outcome relied in part on the chosen computational cutoffs. We observed many partially rescued events when we visualized the AS strength as deltaPSI (dPSI) for all AS events found in both the RNPS1 KD and the RRM or FL rescue data (Supplementary Figure S3B, C). These results suggest that the RRM only incompletely rescues RNPS1-dependent alternatively spliced junctions. We validated this partial rescue with selected transcripts (RER1 and FDPS) using RT-PCR and RT-qPCR in both HEK 293 and HTO cells. Both transcripts are alternatively spliced upon RNPS1 KD. RER1 splicing was largely rescued by RRM expression but splicing of FDPS was still impaired in the HEK293 and HTO cells expressing the RRM (Figure 3B, Supplementary Table S1). This selectivity was also confirmed for two other targets (INTS3 and TAF15), of which INTS3 splicing was rescued, whereas TAF15 was not (Supplementary Figure S3D). Since the RNPS1 RRM is required for ASAP/PSAP assembly, which is also essential for EJC interaction, we speculated that the RRM rescues mainly EJC-dependent splicing events. To this end, we examined exemplary RNPS1-dependent splice events in RNA-Seq data from EJC-protein knockdowns and determined the effects of the RRM rescue. MSTO1 and C5ORF22 are two transcripts with increased AS in the RNPS1 KD, which are also found in EJC KD conditions (Figure 3C, D). While normal splicing of MSTO1 is almost completely restored in RRM-overexpressing cells, the AS event in C5ORF22 remains unchanged in the RRM rescue. Hence, RRM rescue and EJC-dependence do not necessarily correlate. These results indicate that, irrespective of the cell line used for the rescue assay and the EJC-dependence of the splice event, the RNPS1 RRM is able to rescue some, but not all AS events in RNPS1 KD.

### RNPS1 regulates various types of alternative splicing

**Figure 4.**
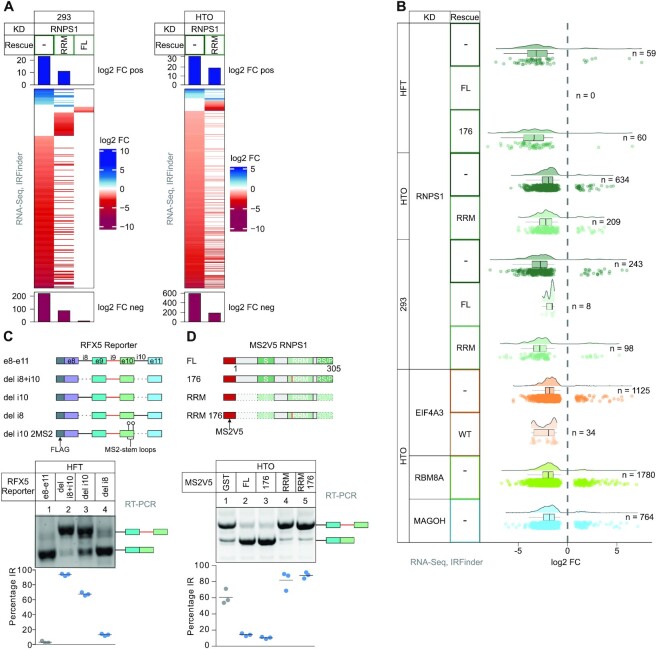
Intron retention events are partially rescued by the RNPS1 RRM. (**A**) log2 fold change (log_2_ FC) of retained introns in the different KD and KD/rescue conditions were determined with IRFinder and are depicted in a Heatmap (Cutoffs: |log2 FC| >1 and *P*adj < 0.001). (**B**) The distribution of log2 fold change (log_2_ FC) of intron retention (IR) events identified by IRFinder is plotted in a raincloud plot. Number of individual events with |log2 FC| >1 and *P*adj < 0.001 is indicated on the right. (**C**) Top: Scheme of RFX5 reporter constructs stably transfected into HFT cells. Bottom: RT-PCR analysis with quantification of intron retention (IR) in the different reporter cell lines, with the resulting PCR-products on the right (*n* = 3). (**D**) Top: RNPS1 tethering constructs transiently co-transfected with the RFX5 del i10 2MS2 reporter into HTO cells. Bottom: IR was analyzed by RT-PCR followed by quantification, PCR-products are indicated on the right (*n* = 3).

RNPS1 was previously shown to also regulate intron retention (IR) events in *D. melanogaster* (24,25). Since LeafCutter is unable to detect IR events, we used the IRFinder algorithm to identify RNPS1-regulated retained introns (58). Overall, the RRM rescued more than half of the RNPS1-dependent IR events in HTO and HEK293 cells (Figure 4A, Supplementary Table S5). However, the splicing of many introns was not rescued at all by the RRM (Figure 4B). The EJC-dependent RFX5 intron 9 retention, for instance, is one of the strongest IR events found in RNPS1 and its splicing was not rescued by the RRM (Supplementary Figure S4A). From a mechanistic point of view, IR is especially interesting, since it represents a seemingly contradictory function of RNPS1: On the one hand, RNPS1 suppresses recursive splicing of cryptic 5′ splice sites, but on the other hand it activates splicing of some introns and thereby represses IR. Therefore, we aimed for a deeper analysis of RFX5 intron 9 splicing. We suspected that splicing of the surrounding introns and subsequent EJC deposition and RNPS1 recruitment reinforces correct RFX5 intron 9 splicing. To test this hypothesis, minigene-reporters in which either one or both introns were deleted were designed and stably transfected into HeLa FT cells (Figure 4C, top). RT-PCR of the different reporters shows that RFX5 intron 9 splicing relies on the splicing of both surrounding introns, but mostly on the subsequent intron 10 (Figure 4C, bottom). This finding matched our hypothesis that EJCs deposited at the surrounding junctions stimulate splicing of intron 9, similar to what has been described for the PIWI pre-mRNA in *D. melanogaster* (24,25). In a tethering assay, a RFX5 reporter in which intron 10 is replaced by MS2 stem loops was co-transfected with different RNPS1 MS2V5-tagged constructs (Figure 4C and D, top). As observed in the RNA-Seq data, the RNPS1 RRM was unable to rescue RFX5 splicing, even in the tethering assay (Figure 4D, bottom). Interestingly, the RNPS1 176 mutant in the full-length context, which cannot assemble ASAP or PSAP and was unable to rescue most RNPS1-dependent AS events, was able to rescue RFX5 intron 9 splicing when tethered to the mRNA. This indicates that IR repression relies on RNPS1 as the effector molecule and does not require complete ASAP/PSAP complexes or EJC recruitment, once RNPS1 is deposited on the mRNA. Although RFX5 correct splicing was not rescued by RNPS1 RRM expression, several IR events were substantially improved, like INTS2 (Supplementary Figure S4B). Therefore, we were wondering whether we can detect discrepancies between RRM rescue of alternative splice sites and of IR. To reveal possible differences, we classified all RNPS1-dependent splice events into categories (exon skipping (ES), alternative 5′ or 3′ splice sites (A5SS/A3SS), exon inclusion (EI) and IR) by using rMATS (49) and determined whether the RRM rescues certain forms of AS. Absolute counts of the various types of AS events and also relative proportions revealed that all types of RNPS1-dependent splicing events were equally well rescued by the RRM (Supplementary Figure S4C, D, Table S6). Taken together, the results of three different bioinformatic analyses show that the RNPS1 KD effects are only partially rescued by the RNPS1 RRM.

### Splicing associated factors are recruited by the RNPS1 RRM

**Figure 5.**
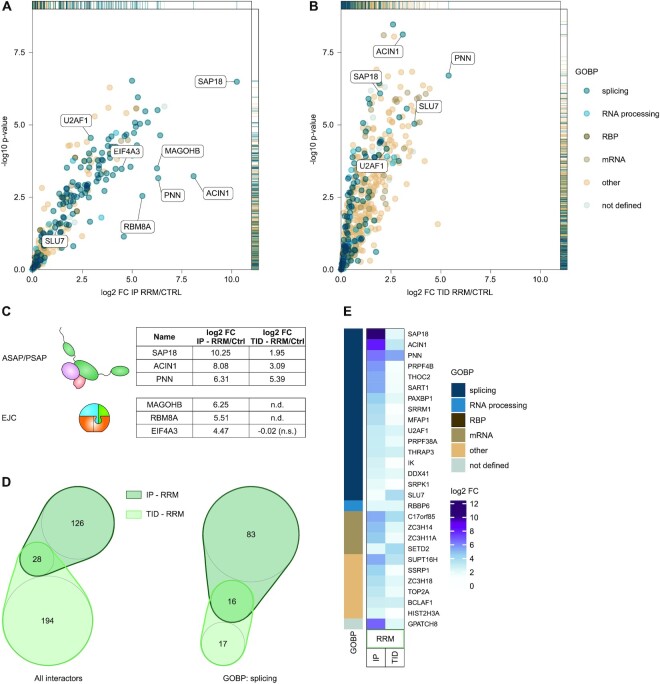
RNPS1 RRM interacts with a broad variety of splicing-regulatory proteins. (**A**) FLAG-RNPS1 RRM construct was overexpressed in HEK293 cells, followed by FLAG-immunoprecipitation (IP) and label-free mass spectrometry (MS). The -log10 *P*-value of identified proteins is plotted against the log_2_ fold change (log_2_ FC) in a volcano plot (cutoff: log_2_ FC ≥ 0). Proteins identified by MS were classified by their gene ontology biological process (GOBP) terms into the main groups splicing, RNA processing, RNA binding protein (RBP), mRNA and other. Proteins without GOBP terms were classified as not defined (Supplementary Tables S7 and S8). (**B**) For TurboID biotin proximity labeling (TID), FLAG-TurboID-tagged RNPS1 RRM was overexpressed in HEK293 cells. Biotinylated proteins were identified via MS after enrichment using streptavidin beads. As in (A), −log_10_*P*-value is plotted against the log_2_ fold change (log2 FC; cutoff: log_2_ FC ≥ 0) in a volcano plot. Proteins were classified as described in (A) using the GOBP terms. (**C**) List of log_2_ fold change (log_2_ FC) of ASAP/PSAP and EJC factors in the IP and in the TurboID (TID) dataset of RNPS1 RRM versus control. (**D**) Venn diagrams display the overlaps of the RNPS1 RRM MS datasets with regard to their GOBP classification (left: all; right: splicing). (**E**) The log_2_ fold change (log_2_ FC) of the overlapping enriched proteins in both RNPS1 RRM MS datasets (IP and TurboID) are depicted in a heatmap and classified (as described in (A)).

Despite the incomplete rescue of RNPS1-dependent AS events, our findings suggest that the RRM can regulate different types of AS events, in addition to the previously shown rescue of cryptic 5′ splice sites (26). Therefore, the RNPS1 RRM presumably assembles a splicing-regulatory complex that mediates at least part of the activity of RNPS1 FL. However, neither the mechanism of cryptic 5′ splice site suppression nor the factors interacting with the RRM (apart from the EJC, ACIN1, PNN and SAP18) are known in detail. To characterize the components of this putative splicing-regulatory complex, we set out to identify protein factors that interact with the RNPS1 RRM. We generated HEK293 cell lines expressing N-terminally FLAG-emGFP-tagged RRM, confirmed its expression by Western blot (WB) and identified co-purified proteins by mass spectrometry (MS) after FLAG-immunoprecipitation from RNase A treated cell lysates (IP; Supplementary Figure S5A). As expected, the RNPS1 RRM efficiently pulled down the three nuclear EJC core components (EIF4A3, RBM8A, MAGOH) and all proteins of the ASAP and PSAP complexes (Figure 5A, Supplementary Tables S7 and S8). Furthermore, RRM-containing complexes contained primarily factors that are involved in splicing or splicing regulation, e.g. U2AF1 and SLU7 (Figure 5A). To confirm the presence of splicing factors in the RRM interactome by an independent method, we fused the RRM of RNPS1 with TurboID (TID) for proximity labeling (36,37). Despite quantitative differences in individual proteins, the RRM interactome determined by TID contained all components of the ASAP and PSAP complex, as well as about 50 proteins with annotated gene ontology biological process (GOBP) terms matching ‘splicing’, ‘RNA processing’, ‘RNA-binding protein’ or ‘mRNA’ (Figure 5B). Notable differences in the TID interactome compared to the IP were: (i) the absence of EJC proteins (Figure 5C); (ii) a lower abundance of SR proteins (Supplementary Tables S7 and S8); (iii) more non-mRNA associated interactors (Figure 5B, D) and (iv) an overall lower fold change enrichment of proteins found in the TID interactome. The lack of EJC enrichment in the TID likely recapitulates the indirect binding of RNPS1 to the EJC, which requires bridging by ASAP/PSAP components. Further possible reasons for the above stated observations could be inherent limitations of TID, caused for example by the absence/low accessibility of lysins in certain proteins or the narrow labeling radius. Notably, of the 28 proteins found in both IP and TID of the RRM, 16 are related to ‘splicing’ and another 4 to ‘mRNA’ (Figure 5D, E). The interaction of these splicing factors with the RRM is in good agreement with the observation that the RRM is sufficient to regulate several specific splicing events. Since the IP revealed many more interacting splicing factors that are not detected in the proximity of the RRM (Figure 5D), indirect recruitment of splicing regulatory proteins e.g. by complex formation, contributes to the overall RRM interactome. In conclusion, although the RRM is not the only splicing-relevant domain of RNPS1, it clearly equips the EJC with splicing regulatory abilities by recruiting splicing-associated factors.

To obtain an indication of whether the splicing regulatory proteins in the core RRM interactome are recruited directly or indirectly, we generated two RRM mutants, in which single or multiple surface-exposed amino acids (21) were mutated (Supplementary Figure S5A). When expressed in RNPS1 depleted cells, both mutations R196E and K203D/Y205D reduced the ability of the RRM to rescue the RNPS1-dependent cryptic splicing of RER1 and INTS3 (Supplementary Figure S5B). However, the same events in RER1 and INTS3 were completely rescued by RNPS1 FL even if the RRM was similarly mutated (Supplementary Figure S5C, D). In contrast, splicing of FDPS and TAF15 could not be rescued by RNPS1 FL carrying the RRM mutations, although both events are not rescued by the RRM alone (Supplementary Figure S5C). This leads to the paradoxical observation that in the full-length context, mutations in the RRM seem to affect events that require other domains of RNPS1 for their correct splicing. To understand the effects of the mutations, we performed FLAG-IPs of the RRM with and without the R196E and K203D/Y205D mutations. As shown by MS and WB analysis, the mutants interacted equally well as the wildtype RRM with the majority of EJC and ASAP/PSAP components (Supplementary Figure S5E-G). However, the K203D/Y205D and to a lesser extent also the R196E, pulled down fewer of the splicing-related factors that are pulled down by the wildtype RRM (Supplementary Figure S5F, G). In summary, we conclude that the RRM of RNPS1 is able to regulate splicing of a subset of splice events by assembling at least partially splicing competent complexes.

### RNPS1 C-terminus mediates interactions with splicing factors and the U1 snRNP

**Figure 6.**
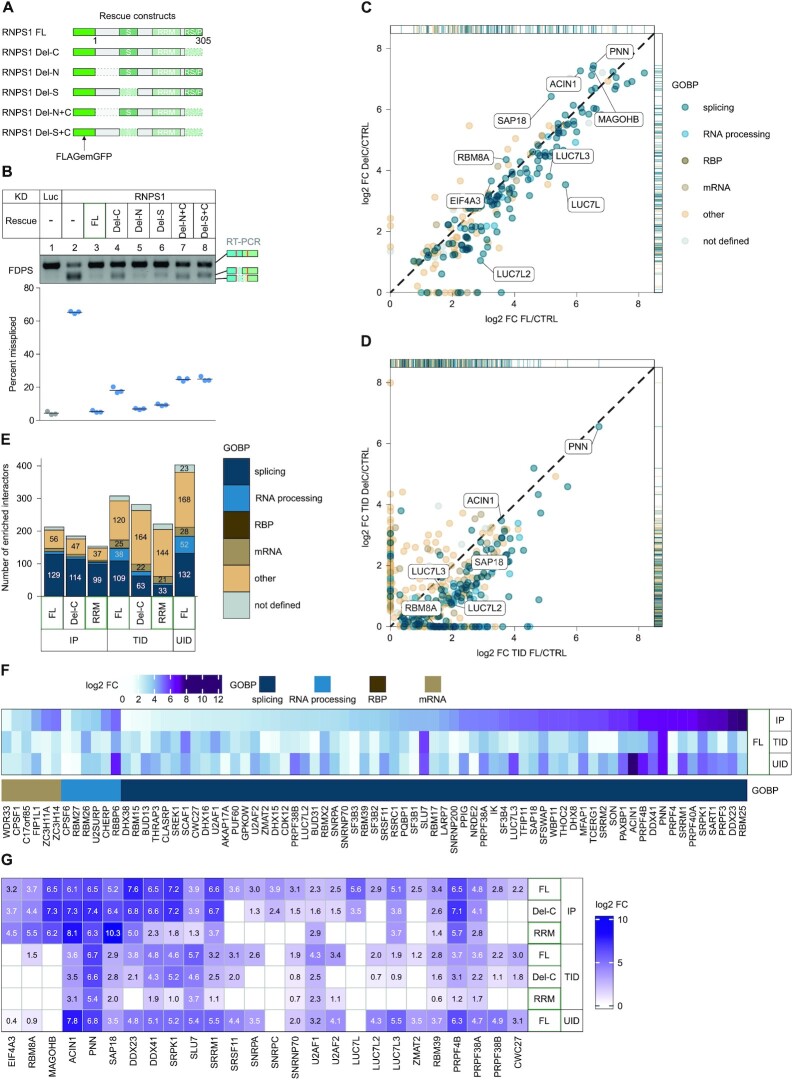
RNPS1 C-terminus is important for regulation of specific alternative splicing events and recruits additional splicing-regulatory proteins. (**A**) Schematic representation of RNPS1 rescue constructs and domain deletions. (**B**) RT-PCR of FDPS alternative splicing (AS) in HEK293 cells after control or RNPS1 KD with expression of the rescue constructs depicted in (A). One representative replicate is shown and quantification is depicted below (*n* = 3). (C, D) RNPS1 full-length RNPS1 (FL) or C-terminally shortened RNPS1 (Del-C) were expressed in HEK293 cells as (**C**) FLAG-emGFP- or (**D**) FLAG-TurboID-tagged constructs. Label free MS was performed using the IP and proximity-labeled (TID) samples. The log_2_ fold change (log2 FC) of proteins enriched in the RNPS1 Del-C was plotted against log_2_ FC of proteins enriched in RNPS1 FL (cutoff: *q*-value < 0.05 in at least one condition). Proteins were classified as described for Figure 5A (Supplementary Tables S7 and S8). (**E**) Bar graph showing the absolute counts of proteins enriched in the different MS datasets according to their GOBP classification (see Figure 5A) (Cutoff: q-value < 0.05 and log2 FC > 1). (**F**) Heatmap of proteins enriched in all three RNPS1 FL datasets (IP, TurboID, UltraID; cutoff: *q*-value < 0.05 and log_2_ FC > 1) that are classified as either splicing, RNA-processing, RBP or mRNA according to their GOBP terms (see Figure 5A). (**G**) log2 FCs in IP, TurboID and UltraID for selected RNPS1 interactors is displayed in a heatmap (cutoff: *q*-value < 0.05). The conditions are indicated on the right side.

The incomplete splicing rescue by the RNPS1 RRM construct clearly demonstrated that other regions of RNPS1 have to play supporting roles in the regulation of certain splicing events. Therefore, we aimed for an in-depth analysis of the N-terminus and the three previously reported functional domains of RNPS1: the so-called S-Domain, the RRM domain and the C-terminal arginine-serine/proline-rich domain (RS/P) (68). We generated various RNPS1 deletion mutants, lacking either one or two of the RNPS1 domains, stably integrated them into the genome of HEK293 cells and induced their expression shortly after RNPS1 KD (Figure 6A, Supplementary Figure S6A). Subsequently, we analyzed if the mutants were able to rescue the RNPS1-dependent AS events in FDPS and TAF15. Both splicing events were fully rescued by RNPS1 FL and the Del-N variant, while the Del-S variant rescues FDPS splicing almost completely but fails to rescue TAF15 splicing (Figure 6B, Supplementary Figure S6B). Deletion of the RNPS1 C-terminus and all combined deletion mutants were unable to rescue TAF15 and FDPS splicing, although the amount of mis-spliced transcript varied between the different mutants. The differences in rescue activity indicated that different domains perform partially redundant functions and are not equally important for the activity of RNPS1. However, there were variations in detail and some splice events appeared to be domain-specific, which we could observe, for example, for the AS of FDPS (Figure 6B, Supplementary Figure S6C). If rescued with the RNPS1 construct lacking the S-domain, an A5SS in exon 4 was used. When the rescue construct lacked the C-terminus, the same A5SS was combined with an A3SS in the fifth exon of FDPS, resulting in a slightly faster migrating band in the gel. In the RNPS1 KD, approximately 30% of the FDPS transcripts resulted from only the A5SS while 50% resulted from alternative 5′ and 3′ splicing and the remaining transcripts were normally spliced. Interestingly, the sashimi plot also shows that the expression of the RRM pushes AS more towards only A5SS usage, similar to the rescue with the Del-S construct (Supplementary Figure S6C).

These experiments have established that in some transcripts, certain domains of RNPS1 are essential for normal splicing and their deletion cannot be compensated by other domains, for example the RRM alone. Overall, the deletion of the C-terminus and the combinatory deletions of the N- and C-terminus or S-domain and C-terminus had the strongest effect on the AS events that we analyzed in detail. Since all three rescue constructs were lacking the C-terminus, we set out to identify proteins that interact with RNPS1 via its C-terminus. We expressed and immunoprecipitated FLAG-emGFP-tagged RNPS1 FL and the C-terminally shortened version (Del-C) and analyzed the interactome by MS. As expected, we found many splicing factors and components of the spliceosome (Supplementary Tables S7 and S8). Notably, several RNPS1-interacting proteins were pulled down less efficiently by RNPS1 Del-C. For example, RNPS1 FL interacted with members of the LUC7-family of splicing factors (LUC7L, LUC7L2, LUC7L3), whose interaction was substantially reduced with RNPS1 Del-C (Figure 6C, Supplementary Tables S7 and S8). The interaction of other splicing factors was also affected by the deletion of the C-terminus. For example, we confirmed the reduced interaction of Del-C with the U1 component SNRPA and splicing factor LUC7L3 by WB (Supplementary Figure S6D). In contrast, no decrease in binding to FL and Del-C RNPS1 was observed for the EJC and the other ASAP-/PSAP components (Figure 6C, Supplementary Figure S6E). We observed a similar effect when we repeated the experiment with a TID proximity labeling assay: splicing-related interactors of RNPS1 were reduced in the interactome of the Del-C mutant, whereas ASAP-/PSAP components remained unchanged (Figure 6D). Similar to the RRM results, we noted some quantitative and qualitative variations between the IP and TID experiments, i.e. whether a certain factor was detected in either or both of the interactomes. Overall, the differences between IP and TID were similar to those we have already described for the RRM interactome (Figure 5A, B). Nevertheless, we also observed, for example, a weaker binding of LUC7 proteins in the TID of RNPS1 Del-C (Figure 6D). When comparing all interactome analyses of RNPS1 FL and its deletion mutants, we clearly noticed the general trend that RNPS1 FL interacts with the highest number of splicing/processing factors and RNA-binding proteins (Figure 6E). Deletion of the C-terminus clearly reduced this number and the RRM alone has the fewest of these proteins in its interactome (Figure 6E). Interestingly, this reduction of splicing-associated factors that interact with the RNPS1 deletion mutants was especially pronounced in the TID. We complemented the two interactome analyses described above with another analysis of UltraID-fused full-length RNPS1 (35). Together, these three datasets enabled us to define a core interactome of RNPS1 of which many proteins have annotated functions in RNA metabolism (Figure 6F). In total, 63 splice factors were found in all three RNPS1 FL interactomes, and another 58 in at least two of them (Figure 6F, Supplementary Figure S6F). Besides the abovementioned proteins of the ASAP/PSAP complex and the LUC7L proteins LU7L2 and LUC7L3, the core interactome also contains U1 snRNP components (SNRNP70, SNRPA), DDX/DHX proteins, components of the spliceosome and RNA-binding proteins (Figure 6F). Some of the core interactors of RNPS1 continued to interact strongly with the Del-C mutant and the RRM (Figure 6G). Others were significantly weakened in their interaction by the deletions or not bound at all (Figure 6G). Altogether, the marked complexity of the RNPS1 FL interactome may reflect the variability of its splicing regulation. Accordingly, the decreased interaction of splicing factors with the Del-C mutant and the RRM correlates with their decreased ability to rescue different splicing events (Figures 3A, 4A and 6G). At this point we are not yet able to assign individual interaction partner to the different functions of RNPS1. However, we speculate that interactions of RNPS1 with the U1 snRNP influence the selection of alternative 5′ splice sites and the suppression of cryptic 5′ splice sites, while the interaction of RNPS1 with U2AF2 affect the choice of 3′ splice sites. These interactions of RNPS1, as well as its ability to bind to the spliceosome and other splicing factors, may also play a role in preventing intron retention.

## DISCUSSION

Although RNPS1 has been the subject of several studies, it remained unclear which of its various functions is most important in the context of the EJC. In this work, we analyze the roles of RNPS1 in NMD and AS regulation and present new molecular details on how AS regulation is mediated by RNPS1. Our analysis of several RNA-Seq datasets suggests that RNPS1 is globally less important for NMD than for example the three core EJC factors EIF4A3, MAGOH or RBM8A. Some NMD targets were upregulated upon RNPS1 depletion, but often only to a low extent (Figure 1D, E and Supplementary Figure S1E). Overexpression of RNPS1, on the other hand, appeared to slightly enhance NMD efficiency. This is in good agreement with previous work in which NMD activation by tethering RNPS1 to reporter mRNAs was shown (62,69). Similarly, the NMD activity of different HeLa cell strains was previously reported to correlate with their RNPS1 expression levels (31). Although the exact mechanism is not known, the NMD-activating function of RNPS1 may result from the formation of complexes containing SR proteins, which are also known to stimulate NMD. Thus, RNPS1 might locally increase the concentration of NMD-promoting proteins on the mRNA, which can lead to a more efficient turnover, but are not absolutely essential for the execution of NMD. However, some recently reported RNPS1-dependent NMD targets exhibited no consistent response to RNPS1 depletion and were either slightly up- or even slightly downregulated in our RNA-Seq datasets (32). Furthermore, only about half of the low number of transcripts (7 of 16) that were identified in the DTU analysis as potential high-confidence NMD isoforms in RNPS1 KD could indeed be observed to be targeted by NMD. Altogether, we conclude that increased RNPS1 expression results in a decreased abundance of specific transcripts, which is in good agreement with an NMD-activating function. In contrast, the RNPS1 KD has globally a rather weak effect on only a low number of NMD-sensitive isoforms.

Since many EJC factors are essential for cellular survival (70), we used siRNA-mediated knockdowns to study their role in NMD. This approach inherently bears the problem of variable and incomplete protein depletion and—in case of essential factors—a potential selection effect for poorly transfected cells. These technical caveats could at least in part be the reason for the sometimes-limited effect size and small overlaps between certain conditions.

Our finding that many of the inspected transcripts identified as RNPS1-dependent NMD targets likely result from AS further emphasizes the importance of splicing regulation by RNPS1. We reported previously that the RRM domain is sufficient to regulate some EJC-dependent splicing events (26). Hence, we started our analysis with the initial hypothesis that the RRM domain is sufficient for the regulation of many, if not all RNPS1-dependent splicing events. Unexpectedly, RNA-Seq analyses of RNPS1-depleted cells rescued with the RRM showed that it can only partially replace RNPS1, both quantitatively and qualitatively. Expression of the RRM frequently resulted in incomplete rescue compared to full-length RNPS1. Many other splice events were not rescued at all by the RRM. However, our attempt to classify all RNPS1-dependent splicing events into RRM-rescued and RRM non-rescued did not yield clear results.

During the detailed analyses of the RNA-Seq datasets, we found the previously described EJC- and RNPS1-dependent splicing events, for example the use of cryptic 5′ and 3′ splice splices (26–29). In addition, we identified multiple examples of introns, which required RNPS1 for efficient splicing. Previous studies in *D. melanogaster* showed that splicing of intron 4 of the PIWI pre-mRNA depends on the deposition of EJCs on exon-exon junctions upstream and downstream (24,25). Our findings demonstrate that this phenomenon is also conserved in human cells. We propose a mechanism similar to that in the fruit fly, namely that the splicing of some weak introns is delayed until the surrounding introns are spliced. Such out-of-order splicing has already been shown for other EJC-dependent splicing events (26). We hypothesize that this splice-activating function of RNPS1 is an important contributor to genome maintenance and prevents inadvertent IR. Mechanistically, multiple EJC-bound RNPS1 proteins appear to cooperate hereby, acting in 3′ and 5′ directions from deposited EJCs.

Our global analysis confirmed that the RRM domain of RNPS1 can rescue different classes of splice events. For this purpose, it interacts with a variety of splicing-related proteins including spliceosomal proteins of different snRNPs. This was an unexpected result, since the main splicing regulatory function of RNPS1 was originally attributed to other domains, whereas the RRM seemed to be involved mainly in the formation of the ASAP or PSAP complex (21). However, our results cannot exclude that some of the splicing-related proteins in the RRM interactome are recruited via the other proteins of the ASAP or PSAP complex. PNN, ACIN1 and SAP18 have all been shown to interact with various splicing factors themselves (71–75). Further analyses will therefore be necessary to identify direct interactions between the individual proteins and to disentangle their precise function.

**Figure 7.**
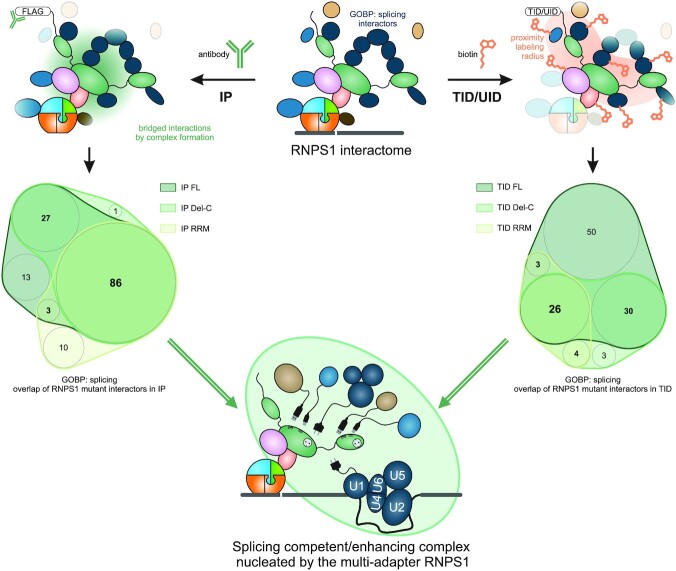
Schematic depiction of splicing competent complexes formed by RNPS1. Top: The RNPS1 interactome was investigated using the orthogonal approaches of IP and proximity labeling (TID = TurboID, UID = UltraID). The Venn diagrams depict in bold large font the RNPS1 core interactome of GOBP splicing labeled proteins that remain in all tested deletion mutants. Bottom: Potential splicing competent/enhancing complex as nucleated by RNPS1, which includes interaction with U1 components and further splicing regulatory proteins.

Although the RRM can assemble a splicing-competent complex, our interactome data clearly show that it can bind only a subset of the splicing factors bound by full-length RNPS1 (Figure 6G). This raises the question how other regions of RNPS1 contribute to splicing regulation. To answer this question, we followed previous classifications of the RNPS1 architecture and examined the function of different deletion mutants (68). The deletion of the S-domain affected some splice events, while deletion of the N-terminus had no effect on the examined events. However, we observed the strongest effects with the deletion of the C-terminus and therefore focused our further analyses on the C-terminal region of RNPS1. Using IP and TID, we show that RNPS1 interacts via its C-terminus with the SNRPA and SNRNP70 components of the U1 snRNP (Figures 6G and 7). Since the U1 snRNP binds to and defines the 5′ splice sites of introns (76), this interaction could be mechanistically involved in the regulation of 5′ splice sites by RNPS1. Interestingly, the suppression of 5′ splice sites was one of the most important functions shown for RNPS1 and the PSAP complex in the context of the EJC (26,27). One seemingly obvious explanation would be that other parts of RNPS1, like the RRM or maybe also the S-domain, repress cryptic 5′ splice sites, while the RNPS1 C-terminus enhances splicing of nearby 5′ splice sites by recruiting the U1 snRNP. Nevertheless, we can only speculate about the exact mechanistic details, and it is also conceivable that the interaction of U1 with the RNPS1 C-terminus prevents cryptic 5′ splicing. Further experiments will be required to unravel the molecular mechanism.

Apart from the interaction with the U1 snRNP, we were able to detect other interactions of RNPS1 and its C-terminus with splice proteins and the spliceosome, for example U2AF1 and U2AF2. Analogous to the interaction with the U1 snRNP, the interaction with U2AF could be used for the regulation of 3′ splice sites, about which not much is known in the context of RNPS1. But it may also play a role in preventing intron retention, either alone or in combination with other interactions. Overall, our interactome analyses indicate that full-length RNPS1 has a more diverse interactome than the Del-C mutant, which itself maintains more interactions than the RRM (Figure 7). Considering previous data and the data from this work, we suggest that RNPS1 bridges splicing factors and spliceosomal components to the EJC, thereby recruiting variable splicing competent complexes to the RNA to guide splicing of nearby introns (Figure 7, Supplementary Figure S7). RNPS1 seems to act as a ‘multi-adapter’ that binds the U1 snRNP, LUC7 family proteins or other splicing factors as required. Due to the amount of different splicing factors that RNPS1 recruits to the pre-mRNA, it can also regulate several different AS events (Supplementary Figure S7). The specific way in which RNPS1 acts on each splicing event is determined by the context and the exact position of the EJC. For example, if there are poorly defined introns in its vicinity, RNPS1 stimulates their splicing. In the case of cryptic 5′ splice sites located downstream of RNPS1, it helps to define exonic regions of the mRNA and prevent their re-splicing. The formation of splice-supporting, high-molecular-weight complexes can best explain the role of RNPS1 and could also serve as a model for the mechanism of other multifunctional splicing factors. Moreover, it also fits well with the higher-order mRNP complexes described in the context of nuclear EJC-bound mRNAs (32). Although our model mainly considers the function of RNPS1 in the context of the EJC, it is possible that it can also bind directly to mRNA or is recruited by some of the above-mentioned proteins to mRNA. This would also explain why not all splicing events regulated by RNPS1 are also EJC-dependent. Interestingly, we had previously observed that the C-terminus can interact nonspecifically with RNA (26). This could reflect its multiple interactions with other RNA-binding proteins, or indicate an intrinsic affinity for RNA.

RNPS1 is of great interest as a multifunctional splicing protein, because it can either suppress or activate splice sites and enhance the splicing of weak introns, which might seem contradictory at first (Supplementary Figure S7). It carries out these functions in conjunction with various other proteins, especially the EJC. This network of interactions allows RNPS1 to regulate a variety of AS events, as it does not rely on its own RNA-binding ability, unlike SR proteins for example. Thus, RNPS1 could be the prototype of flexible, sequence-independent splice regulators, which can be used in regions where no other splicing enhancers can be present due to evolutionary constraints. It will be interesting to find out if other splicing factors can work in a similar way. On the other hand, the interaction of RNPS1 with the EJC needs to be characterized in more detail. So far, we only have some indications how the ASAP complex might interact with the EJC, but more insights will be needed to better understand the 3D structure of EJC-ASAP or EJC-PSAP assemblies. This would also allow us to understand their effect on adjacent splice sites and introns.

## DATA AVAILABILITY

RNA-sequencing data generated for this manuscript have been deposited in the ArrayExpress database at EMBL-EBI (www.ebi.ac.uk/arrayexpress) under accession number E-MTAB-10768 [https://www.ebi.ac.uk/arrayexpress/experiments/E-MTAB-10768] for the RNPS1 HTO dataset, accession number E-MTAB-10770 [https://www.ebi.ac.uk/arrayexpress/experiments/E-MTAB-10770] for the RNPS1 HEK 293 dataset and accession number E-MTAB-10771 [https://www.ebi.ac.uk/arrayexpress/experiments/E-MTAB-10771] for the EJC HTO dataset.

Published datasets analysed for this paper include: ArrayExpress accession number E-MTAB-6564 [https://www.ebi.ac.uk/arrayexpress/experiments/E-MTAB-6564] for the RNPS1 HeLa FT dataset (26) and ArrayExpress accession number E-MTAB-9330 [https://www.ebi.ac.uk/arrayexpress/experiments/E-MTAB-9330] for the SMG7 KO and SMG6 KD HEK 293 dataset (34).

The mass spectrometry proteomics data have been deposited to the ProteomeXchange Consortium via the PRIDE (77) partner repository with the dataset identifier PXD027251 [https://www.ebi.ac.uk/pride/archive/projects/PXD027251] for the co-immunoprecipitation experiments and with the dataset identifier PXD032952 [https://www.ebi.ac.uk/pride/archive/projects/PXD032952] for the proximity labeling experiments.

Published protein structure of the ASAP complex was used (PDB: 4A8X [http://doi.org/10.2210/pdb4A8X/pdb]) (21).

All relevant data supporting the key findings of this study are available within the article and its Supplementary files or from the corresponding author upon reasonable request.

## Supplementary Material

gkac428_Supplemental_Files

## Notes

Present address: Janine Altmüller, Berlin Institute of Health at Charité – Universitätsmedizin Berlin, Core Facility Genomics, Charitéplatz 1, 10117 Berlin, Germany and Max Delbrück Center for Molecular Medicine in the Helmholtz Association (MDC), Berlin, Germany.
