# Supplementary material for: Exon junction complex-associated multi-adapter RNPS1 nucleates splicing regulatory complexes to maintain transcriptome surveillance
Source: Nucleic Acids Res. 2022 May 30;50(10):5899–918. doi: 10.1093/nar/gkac428 (PMC9178013; doi:10.1093/nar/gkac428)
Supplement: gkac428_Supplemental_Files [file gkac428_supplemental_files.zip › Schlautmann_et_al_2022_Supplementary_Figures.pdf]

<u>Supplementary Figures</u>

Exon junction complex-associated multi-adapter RNPS1 nucleates splicing regulatory complexes to maintain transcriptome surveillance

Lena P. Schlautmann[1,2], Jan-Wilm Lackmann[3], Janine Altmüller[4,5], Christoph Dieterich[6,7], Volker Boehm[1,2], Niels H. Gehring[1,2,*]

[1] Institute for Genetics, University of Cologne, 50674 Cologne, Germany
[2] Center for Molecular Medicine Cologne (CMMC), University of Cologne, 50937 Cologne, Germany
[3] CECAD Research Center, University of Cologne, Joseph-Stelzmann-Str. 26, 50931 Cologne, Germany
[4] Cologne Center for Genomics (CCG), University of Cologne, 50931 Cologne, Germany
[5] Present address: Berlin Institute of Health at Charité – Universitätsmedizin Berlin, Core Facility Genomics, Charitéplatz 1, 10117 Berlin, Germany and Max Delbrück Center for Molecular Medicine in the Helmholtz Association (MDC), Berlin, Germany
[6] Section of Bioinformatics and Systems Cardiology, Department of Internal Medicine III and Klaus Tschira Institute for Integrative Computational Cardiology, Heidelberg University Hospital, 69120 Heidelberg, Germany
[7] DZHK (German Centre for Cardiovascular Research), Partner site Heidelberg/Mannheim, 69120 Heidelberg, Germany

*Contact: Niels H. Gehring, ngehring@uni-koeln.de

This PDF file includes:

Supplementary Figure 1 to 7

Schlautmann et al. 2022 - Supplementary Figure 1

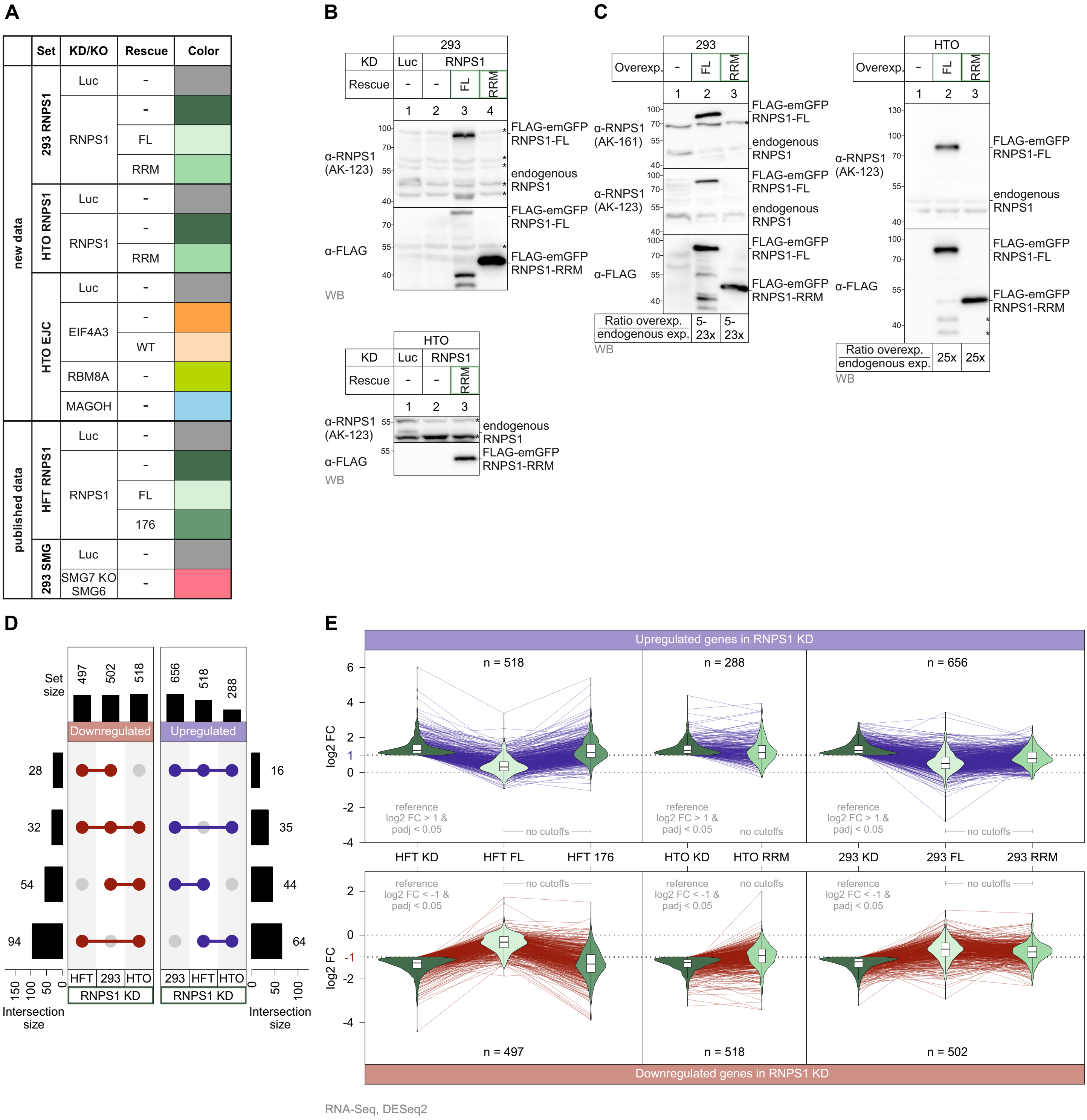

Supplementary Figure 1: Characterization of RNPS1 knockdown conditions.
(A) Overview of published and newly generated RNA-Sequencing (RNA-Seq) datasets, indicating which human cell lines, siRNA-mediated knockdown (KD), CRISPR knockout (KO) and, if applicable, rescue construct were employed. Each condition is assigned to a specific color that is used throughout this manuscript.
(B) Western blots (WBs) of RNPS1 KD and FLAG-emGPF-tagged full-length RNPS1 (FL) or RNPS1 RRM rescue in HTO and HEK293 cells. Antibodies used are shown on the left and a representative replicate is shown (n=3).
(C) WBs of RNPS1 overexpression in HEK293 and HTO cells was analyzed with two antibodies and overexpression compared to endogenous RNPS1 levels was calculated.
(D) Overlap of up- and downregulated genes among RNPS1 KDs in the three different cell types as calculated by DESeq2 (Cutoffs: adjusted p-value (padj) < 0.05 and |log2 FC| > 1).
(E) Combined violin and parallel coordinate plots show genes up- or downregulated upon RNPS KD or rescue with the indicated constructs. Only DGE events that are found in the RNPS1 KD conditions with the indicated cutoffs are plotted, no cutoffs were applied to the other conditions..

Schlautmann et al. 2022 - Supplementary Figure 2

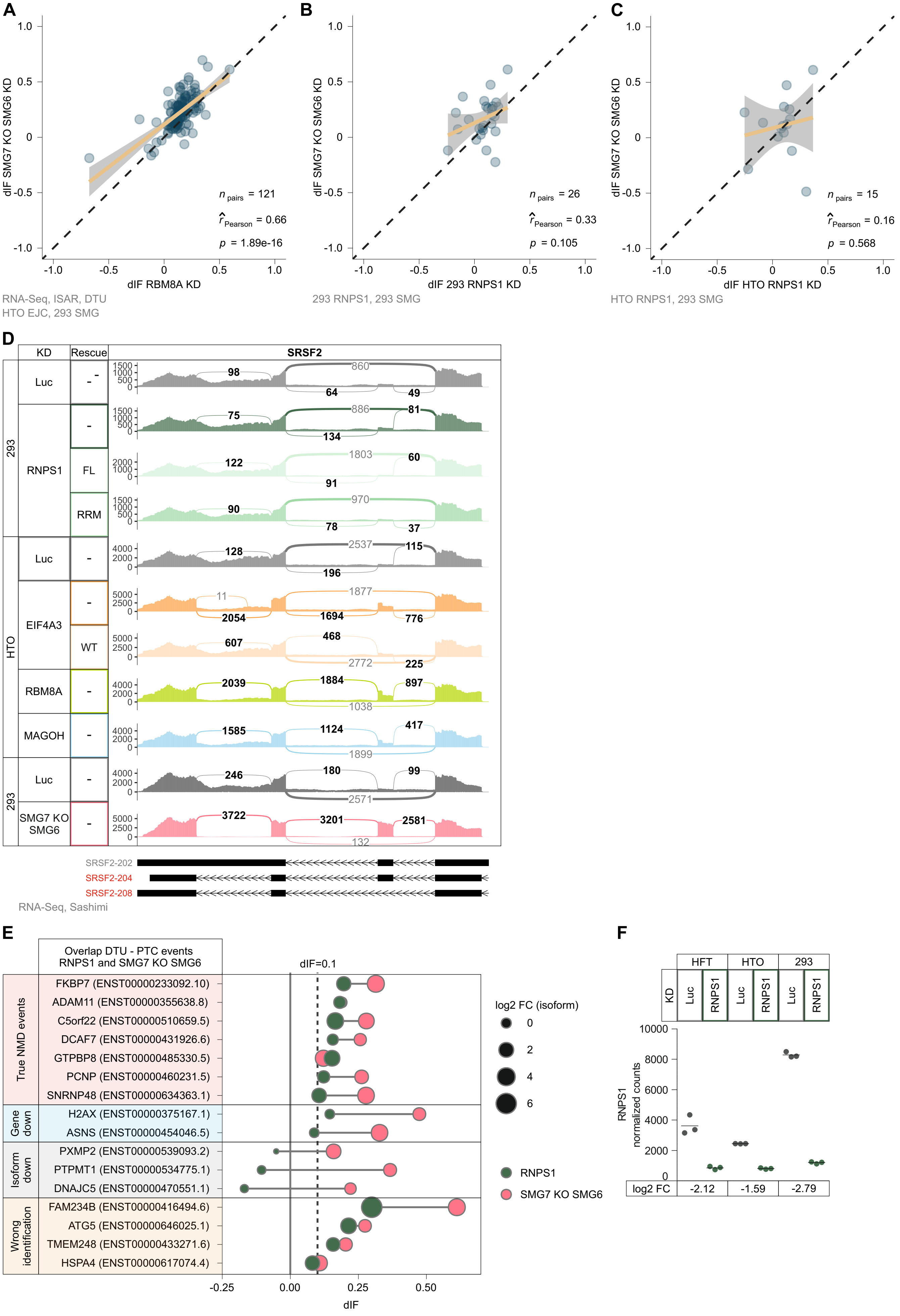

Supplementary Figure 2: Differential transcript usage does not correlate well between RNPS1 KD and SMG7 and SMG6 depleted cells.
(A, B, C) Scatter plots of differential transcript usage (DTU; cutoff: padj < 0.001) in the indicated RNA-Seq data as calculated by IsoformSwitchAnalyzeR (ISAR). npairs is the number of differentially used transcripts found in both conditions, rPearson is the Pearson correlation coefficient and p is the corresponding p-value. (A) RBM8A KD compared to SMG7 KO SMG6 KD, (B) RNPS1 KD in HEK293 cells compared to SMG7 KO SMG6 KD, (C) RNPS1 KD in HTO cells compared to SMG7 KO SMG6 KD.
(D) Mean junction coverage of the NMD-relevant junctions in SRSF2 in the indicated RNA-Seq KD and KD/rescue conditions is displayed as sashimi plot. Canonical (black) and NMD-relevant (red) isoforms are depicted below.
(E) The dIF of the 16 genes that were overlapping in RNPS1 KD and SMG7 KO + SMG6 KD according to ISAR were plotted for the two conditions (Cutoffs for SMG7 KO + SMG6 KD: PTC = TRUE & dIF > 0.1 & adj. p-value < 0.001). Circle size depicts log2 FC of the respective isoform and KD/KO conditions are color-coded.
(F) Expression levels of RNPS1 in control compared to KD conditions in the different cell types plotted as normalized counts. log2 FC of each RNPS1 KD to control are indicated at the bottom.

Schlautmann et al. 2022 - Supplementary Figure 3

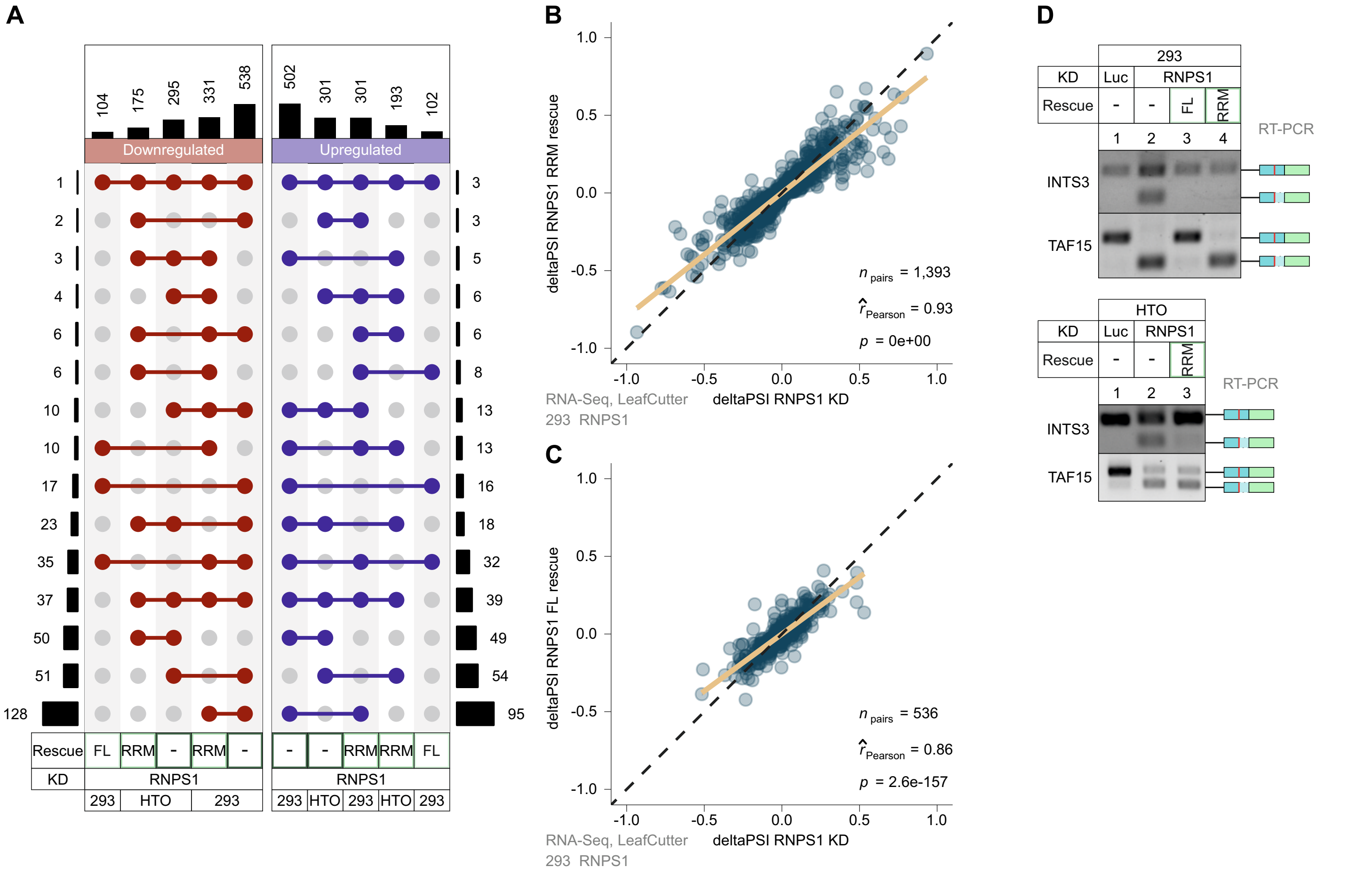

Supplementary Figure 3: RNPS1 RRM rescue of alternative splicing events is incomplete
(A) The intersections of the LeafCutter alternative splicing analysis between the different RNPS1 RNA-Seq sets is shown as an Upset plot (|deltapsi| > 0.1 & padj < 0.001).
(B, C) Scatter plots of alternative splicing (AS) events detected by LeafCutter (Cutoff: padj < 0.001) that are found in both of the indicated conditions in HEK 293 cells. npairs is the number of alternative splicing events found in both conditions, rPearson is the Pearson correlation coefficient and p is the corresponding p-value. (B) RNPS1 RRM rescue compared to RNPS1 KD, (C) RNPS1 FL rescue compared to RNPS1 KD.
(D) INTS3 and TAF15 AS events in RNPS1 KD and RNPS1 FL or RRM rescue were analyzed by RT-PCR. The resulting AS products are indicated on the right and a representative replicate is shown (n=3).

Schlautmann et al. 2022 - Supplementary Figure 4

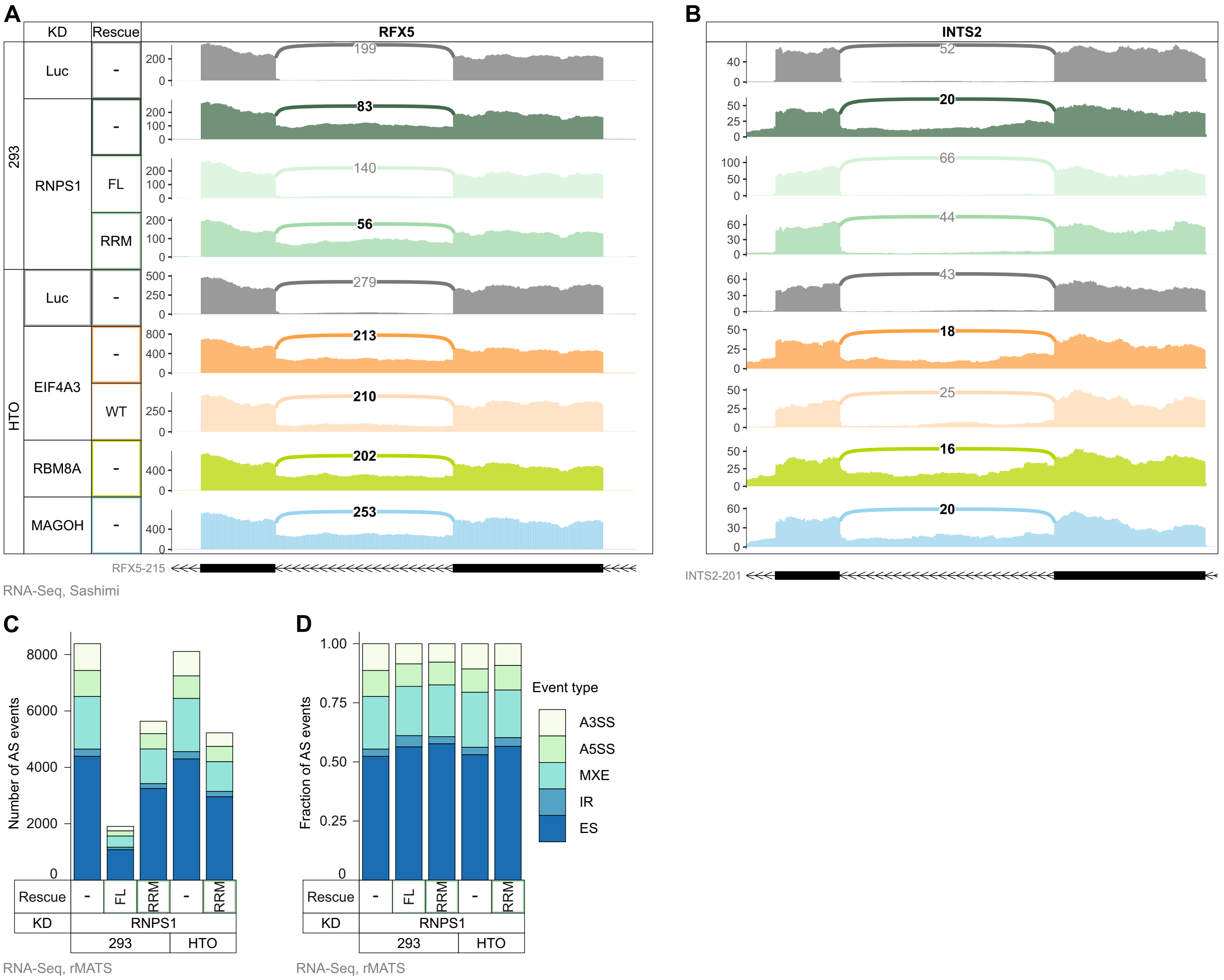

Supplementary Figure 4: RNPS1 RRM rescues alternative splicing irrespectively of the underlying alternative splicing type.
(A, B) RFX5 (A) and INTS2 (B) mean junction coverage across the potentially retained intron in the different RNA-Seq datasets as Sashimi plot. Relevant alternatively spliced junction reads are highlighted.
(C, D) AS event types as detected by rMATS in HEK 293 and HTO RNA-Seq data (Cutoffs: |dPSI| > 0.2 & padj < 0.01). (C) Absolute counts, (D) Relative fractions.

Schlautmann et al. 2022 - Supplementary Figure 5

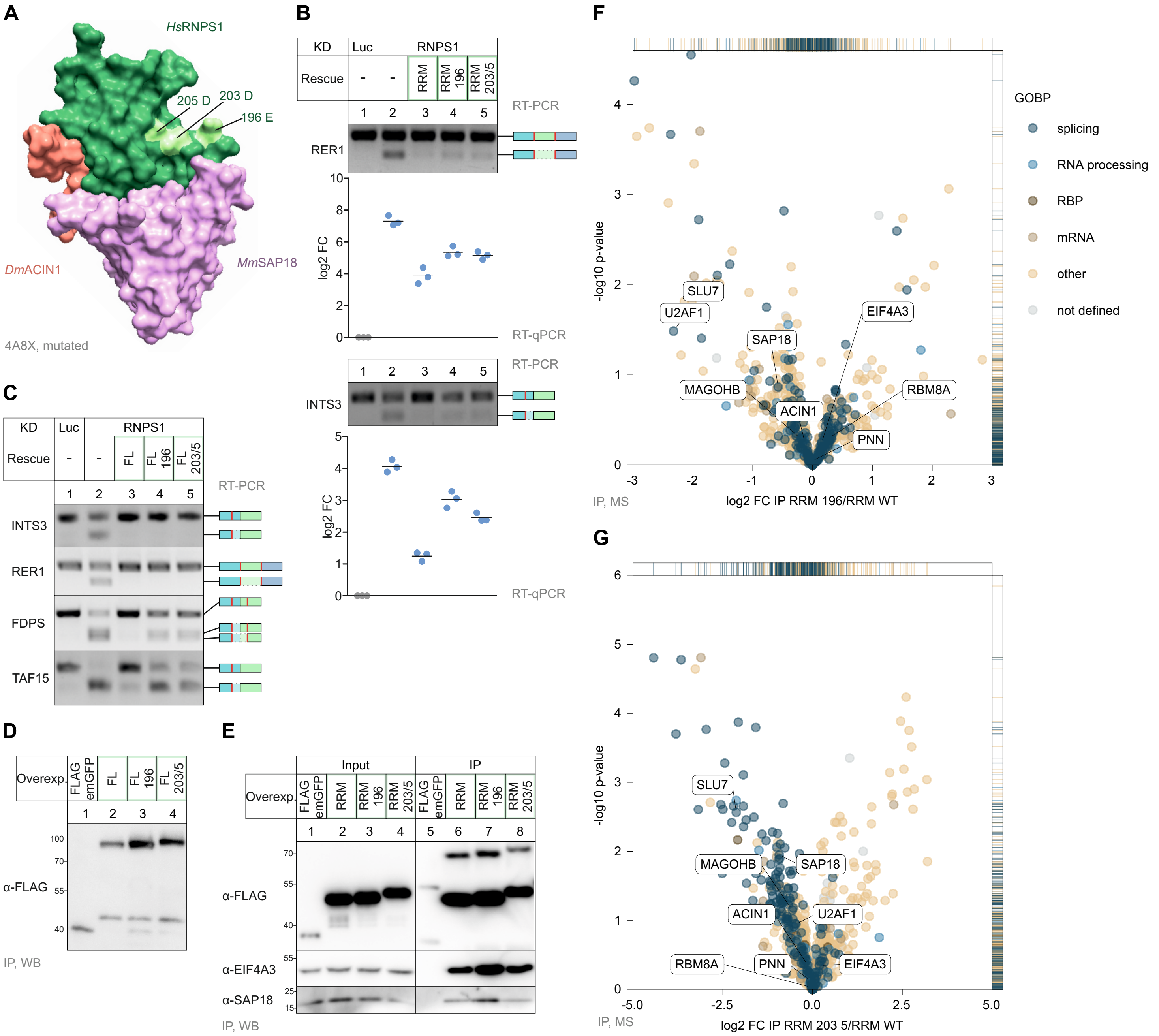

Supplementary Figure 5: Mutations in the RNPS1 RRM reduce its ability to rescue alternative splicing events.
(A) Structure of the ASAP complex with the indicated RNPS1 mutated residues highlighted in light green (PDB accession number 4A8X,).
(B) RT-PCR and RT-qPCR of RER1 and INTS3 from HEK 293 cells exposed to control or RNPS1 KD and expressing the indicated rescue construct. RT-PCR was performed in triplicates (n=3), one representative replicate is shown and the resulting PCR-product is depicted on the right. For RT-qPCR, the log2 FC of the alternatively spliced transcript to the normal transcript is calculated and plotted as datapoints and means (n=3).
(C) RT-PCR of INTS3, RER1, FDPS and TAF15 was performed in triplicates (n=3) in the indicated KDs and KD/rescues. One representative replicate is shown with the resulting PCR product depicted on the right.
(D) WB with anti-FLAG antibody to detect the expression of RNPS1 point-mutants in full-length context (n=3).
(E) WB showing the co-immunoprecipitation of the exon junction complex (EJC) component EIF4A3 and ASAP/PSAP component SAP18 by RNPS1 RRM and the RRM 196 and RRM 203/5 mutants (n=3).
(F, G) -log10 p-value of FLAG-IP mass spectrometry (MS) plotted against log2 FC in a volcano plot for (F) RRM 196 mutant or (G) RRM 203/205 compared to the unmutated RRM.

Schlautmann et al. 2022 - Supplementary Figure 6

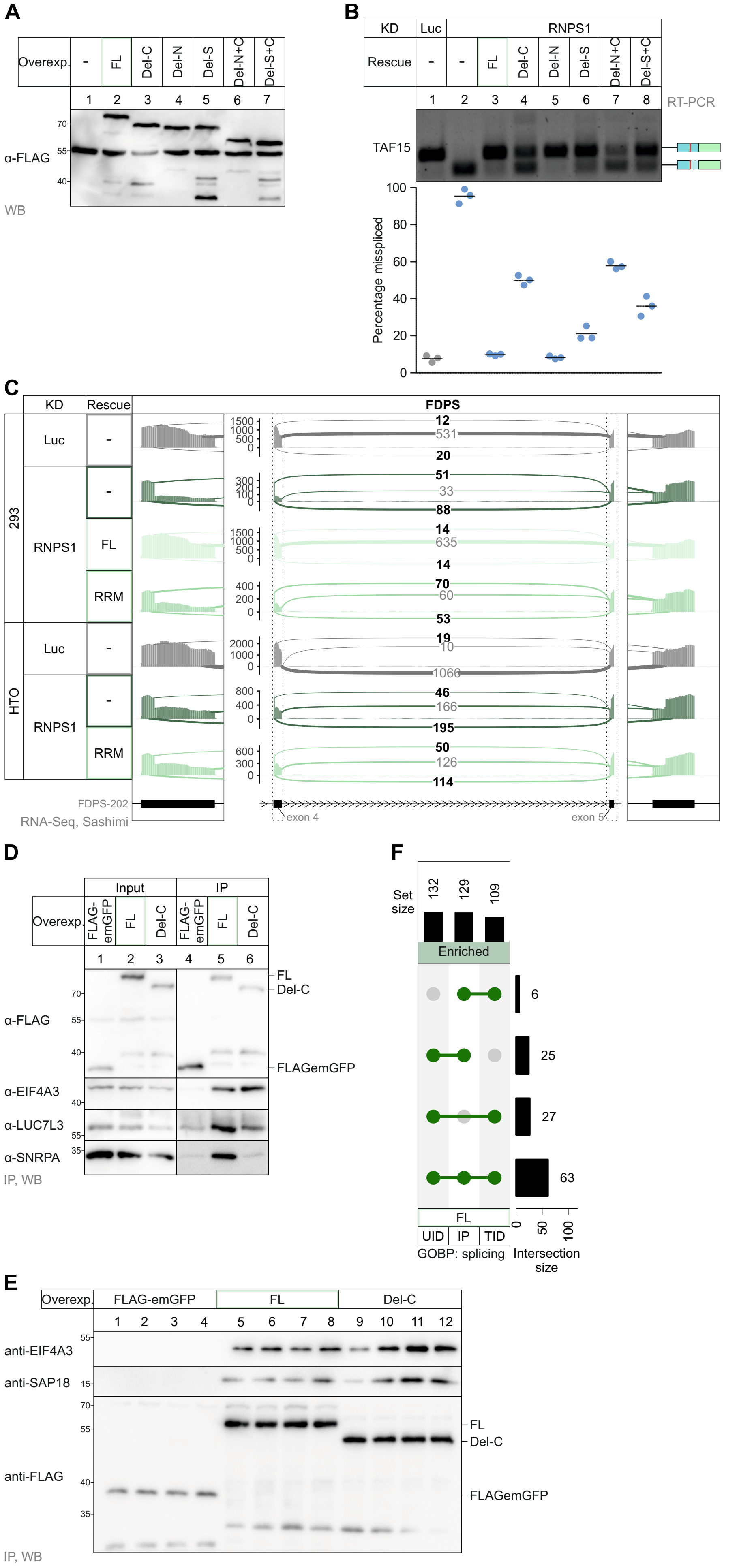

Supplementary Figure 6: Characterization of RNPS1 deletion mutants.
(A) Expression of RNPS1 deletion mutants was validated using WB with anti-FLAG antibody (n=1).
(B) RT-PCR of TAF15 AS in HEK 293 cells after control or RNPS1 KD with the rescue constructs depicted in (A). One representative replicate is shown (n=3).
(C) FDPS RNA-Seq mean junction coverage is shown as sashimi plots with alternatively spliced junction reads highlighted.
(D) FLAG-IP of control, RNPS1 FL and RNPS1 Del-C is analyzed by WB for co-precipitation of EIF4A3 and SAP18, an EJC or ASAP/PSAP component, respectively (n=3).
(E) FLAG-IPs of HEK 293 cells overexpressing either a CTRL, RNPS1 FL or RNPS1 Del-C analyzed by Western blot (WB). Antibodies used are shown on the left and a representative replicate is shown (n=3).
(F) The Upset plot shows the overlaps between the three different RNPS1 FL MS datasets with the GOBP term splicing (Cutoff: q-value <0.05 and log2 FC >1).

Schlautmann et al. 2022 - Supplementary Figure 7

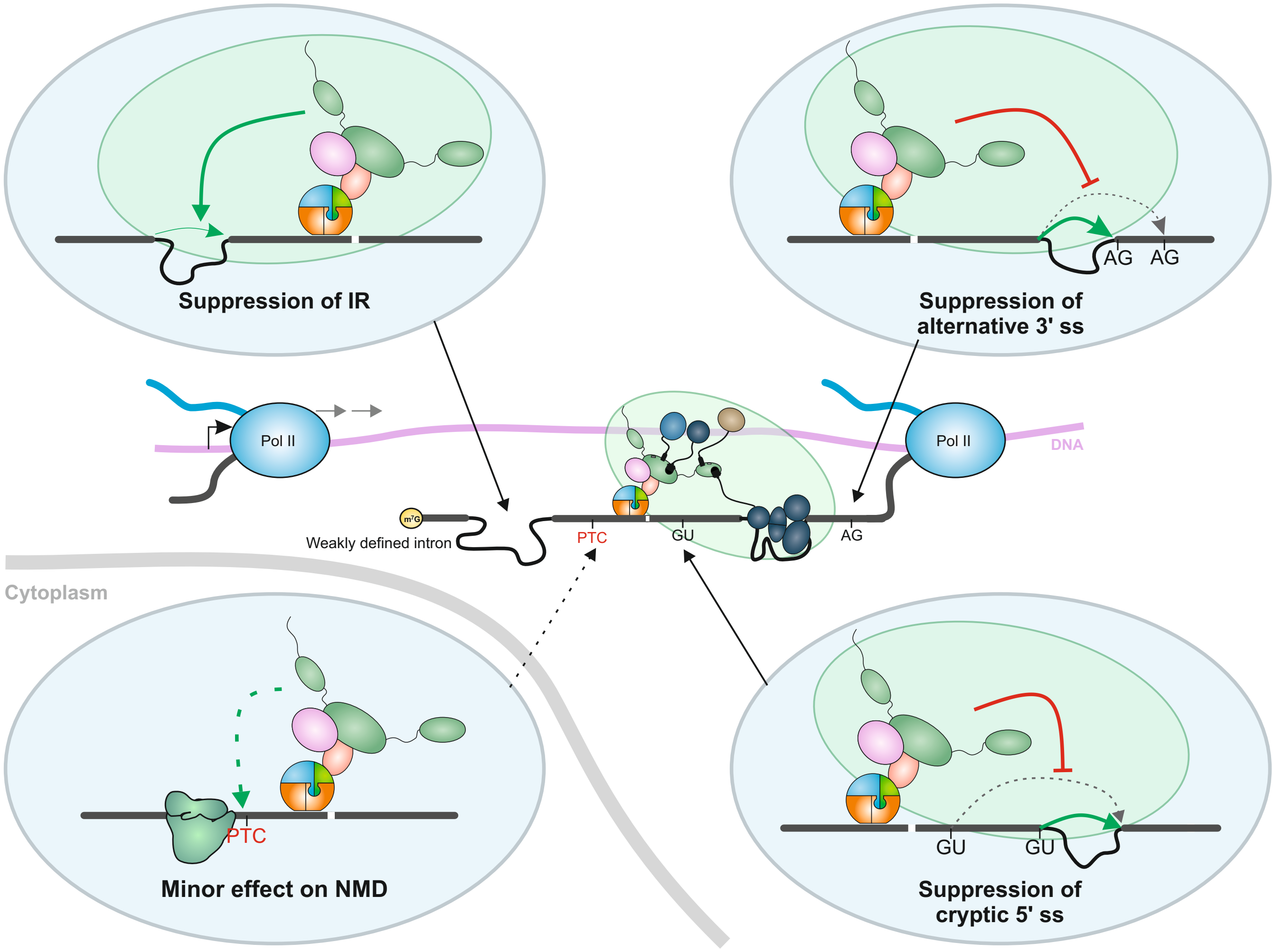

Supplementary Figure 7: Model for alternative splicing regulation by RNPS1.
Correct splicing of not-well defined introns requires EJC deposition and RNPS1 recruitment via ASAP/PSAP. By assembling a splicing competent or splicing enhancing complex, RNPS1 prevents IR and represses alternative 3′ and alternative 5′ splice sites. NMD is mildly activated if RNPS1 is bound to an mRNA.
