# Supplementary material for: Exon junction complex-associated multi-adapter RNPS1 nucleates splicing regulatory complexes to maintain transcriptome surveillance
Source: Nucleic Acids Res. 2022 May 30;50(10):5899–918. doi: 10.1093/nar/gkac428 (PMC9178013; doi:10.1093/nar/gkac428)

Supplementary Dataset 1

Exon junction complex-associated multi-adapter RNPS1 nucleates splicing regulatory complexes to maintain transcriptome surveillance

Lena P. Schlautmann[1,2], Jan-Wilm Lackmann[3], Janine Altmüller[4,5], Christoph Dieterich[6,7], Volker Boehm[1,2], Niels H. Gehring[1,2,*]

[1] Institute for Genetics, University of Cologne, 50674 Cologne, Germany
[2] Center for Molecular Medicine Cologne (CMMC), University of Cologne, 50937 Cologne, Germany

[3] CECAD Research Center, University of Cologne, Joseph-Stelzmann-Str. 26, 50931 Cologne, Germany

[4] Cologne Center for Genomics (CCG), University of Cologne, 50931 Cologne, Germany
[5] Present address: Berlin Institute of Health at Charité – Universitätsmedizin Berlin, Core Facility Genomics, Charitéplatz 1, 10117 Berlin, Germany and Max Delbrück Center for Molecular Medicine in the Helmholtz Association (MDC), Berlin, Germany

[6] Section of Bioinformatics and Systems Cardiology, Department of Internal Medicine III and Klaus Tschira Institute for Integrative Computational Cardiology, Heidelberg University Hospital, 69120 Heidelberg, Germany

[7] DZHK (German Centre for Cardiovascular Research), Partner site Heidelberg/Mannheim, 69120 Heidelberg, Germany

*Contact: Niels H. Gehring, ngehring@uni-koeln.de

	Transcripts	RNPS1 KD - NMD relevant	Comment
**FKBP7**	Unspliced<br><br>Transcript isoforms:<br><br>Non-NMD isoform<br>(ENST00000424785.7)<br><br>NMD isoform(s) in SMG6/7 KD/KO<br>(ENST00000233092.10) PTC<br><br>Relevant isoform(s) in RNPS1 KD<br>(ENST00000233092.10) PTC	✓	One of the FKBP7 NMD sensitive isoforms is upregulated in RNPS1 KD<br>Reason: exon inclusion

The isoform switch in FKBP7 (control vs RNPS1_KD)

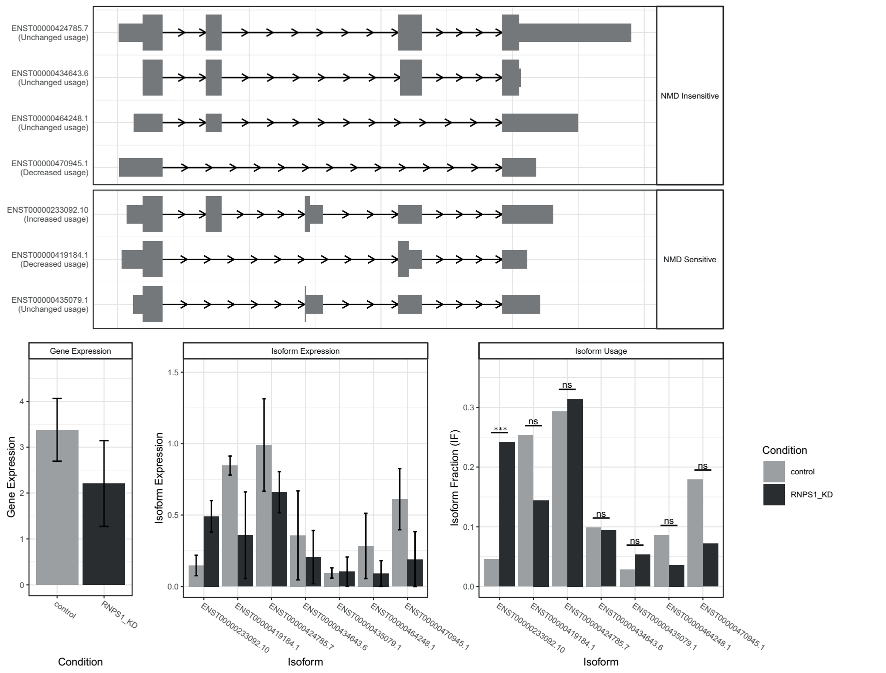

The isoform switch in FKBP7 (control vs SMG7_KO_2_SMG6_KD)

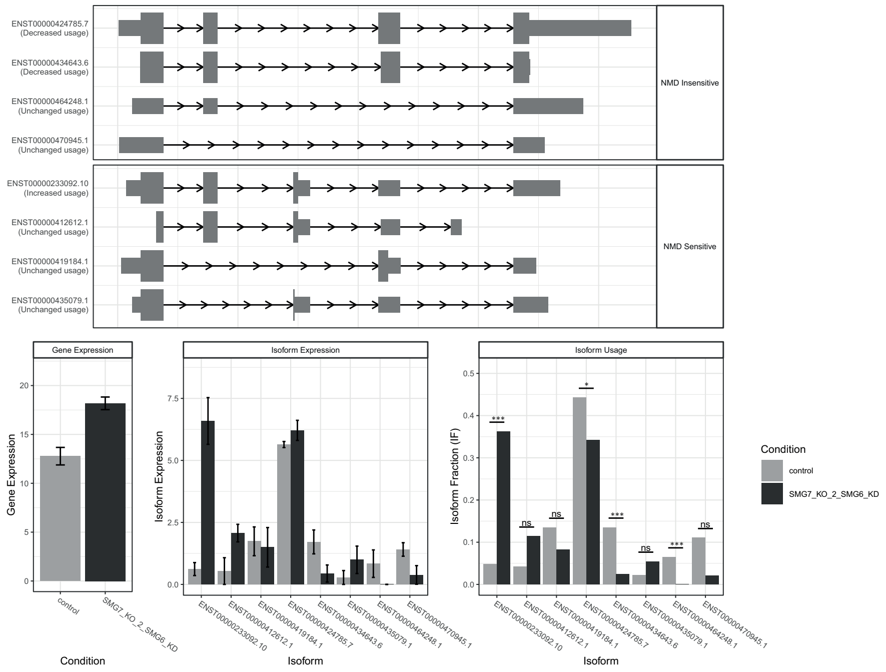

	Transcripts	RNPS1 KD - NMD relevant	Comment

ADAM11

Unspliced

Transcript isoforms:

Non-NMD isoform
(ENST00000200557.11)

NMD isoform(s) in SMG6/7 KD/KO
(ENST00000355638.8)

Relevant isoform(s) in RNPS1 KD
(ENST00000355638.8)

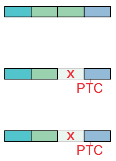

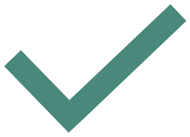

ADAM11 NMD sensitive isoform is upregulated in RNPS1 KD
Reason: alternative 5' SS

The isoform switch in ADAM11 (control vs RNPS1_KD)

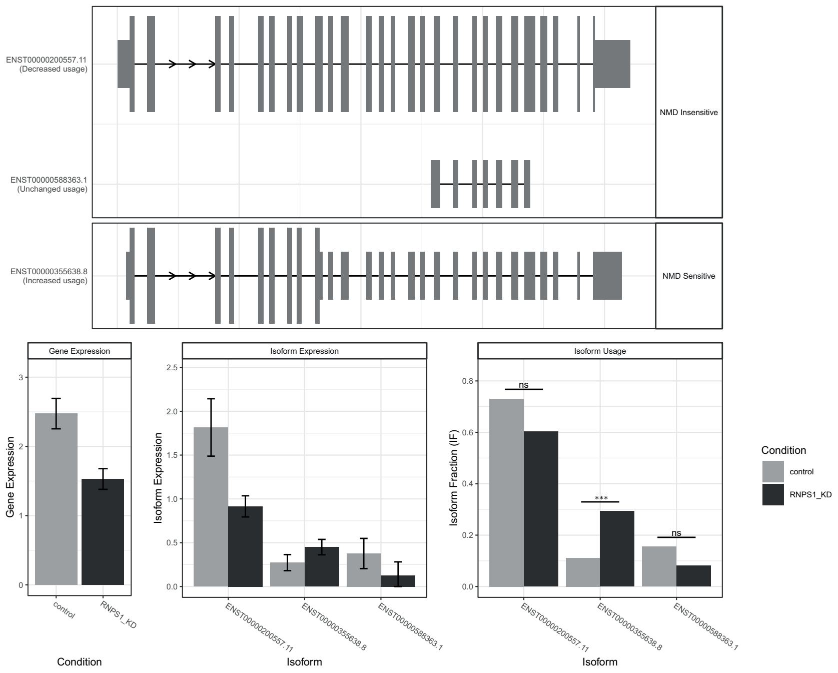

The isoform switch in ADAM11 (control vs SMG7_KO_2_SMG6_KD)

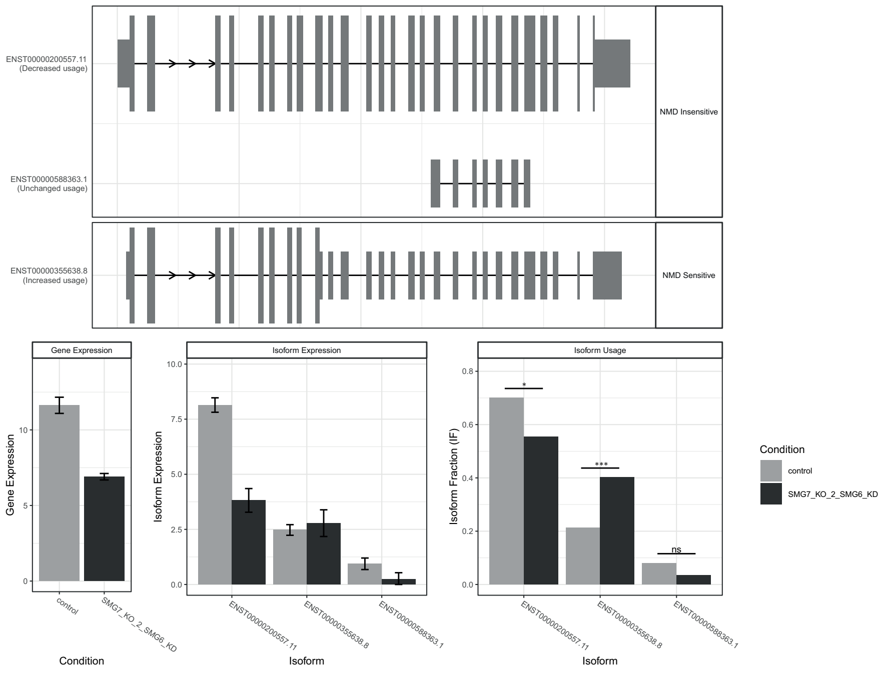

	Transcripts	RNPS1 KD - NMD relevant	Comment
**C5orf22**	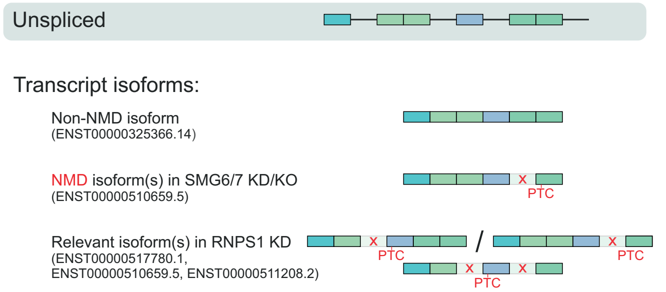	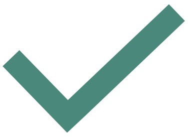	In addition to the NMD-inducing usage of an A3SS, in RNPS1 KD also an A5SS is used. All events, either alone or in combination are predicted to induce NMD

The isoform switch in C5orf22 (control vs RNPS1_KD)

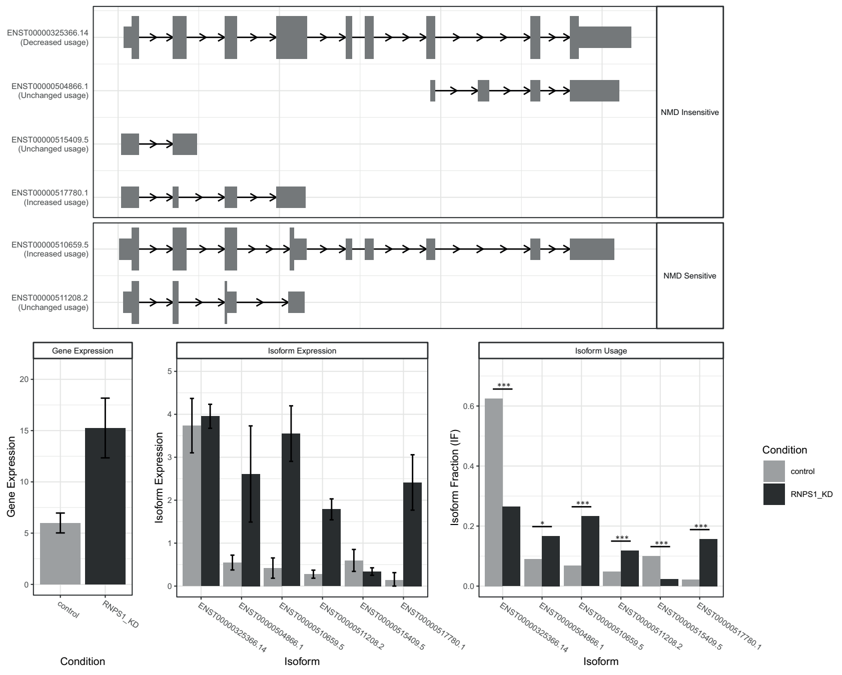

The isoform switch in C5orf22 (control vs SMG7_KO_2_SMG6_KD)

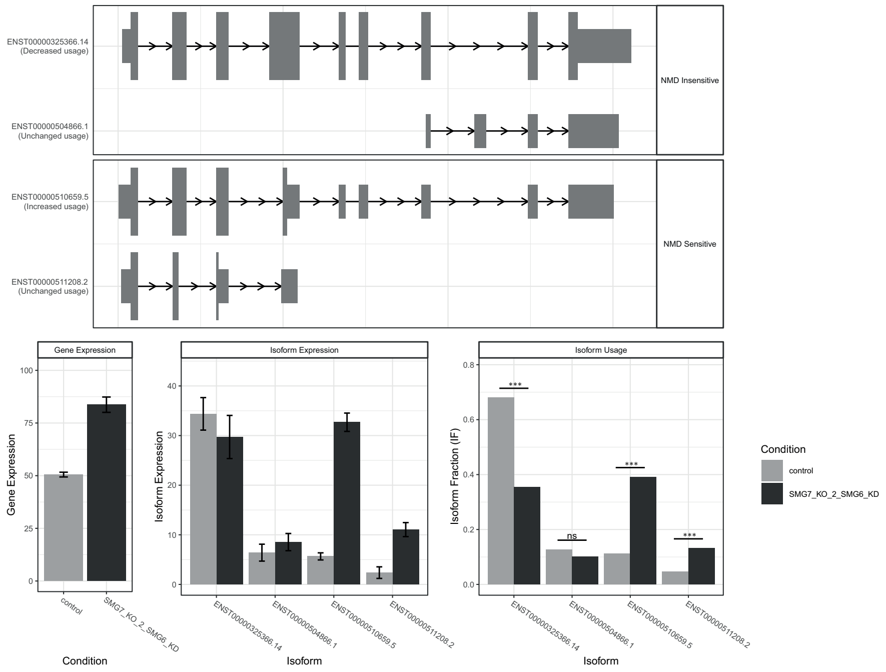

	Transcripts	RNPS1 KD - NMD relevant	Comment
**DCAF7**	Unspliced (STOP)<br><br>Transcript isoforms:<br><br>Non-NMD isoform (ENST00000614556.5) — STOP<br><br>NMD isoform(s) in SMG6/7 KD/KO (ENST00000431926.6) — X, STOP<br><br>Relevant isoform(s) in RNPS1 KD (ENST00000431926.6) — X, STOP	✓	DCAF7 NMD sensitive isoform is upregulated in RNPS1 KD<br>Reason: 3' UTR splicing

The isoform switch in DCAF7 (control vs RNPS1_KD)

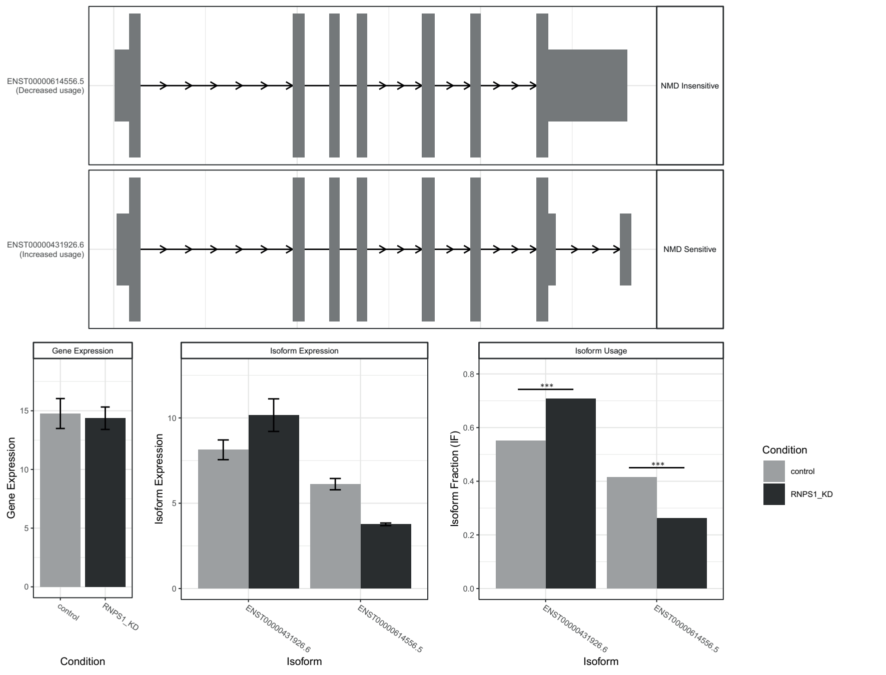

The isoform switch in DCAF7 (control vs SMG7_KO_2_SMG6_KD)

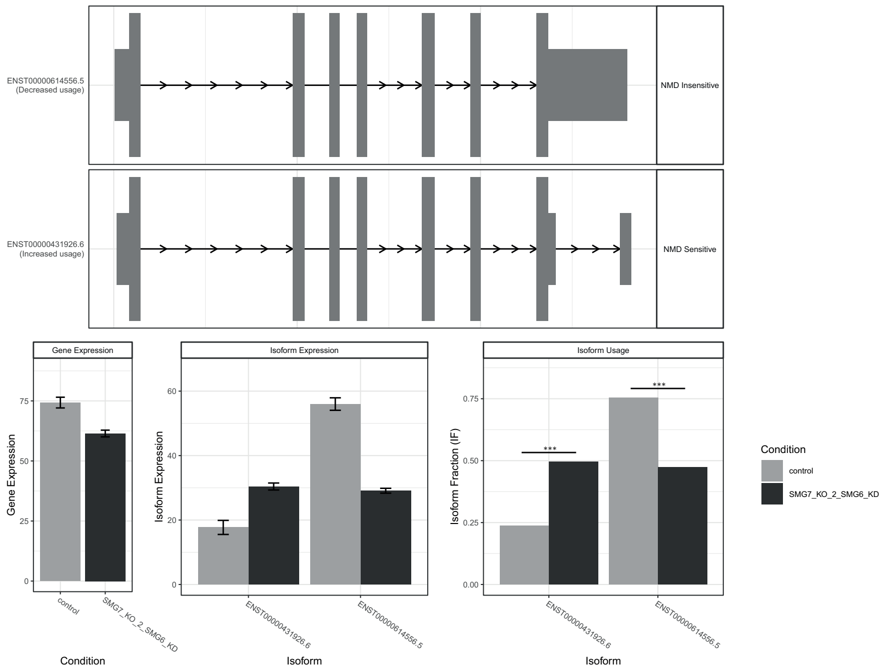

	Transcripts	RNPS1 KD - NMD relevant	Comment
**GTPBP8**	Unspliced<br><br>Transcript isoforms:<br>Non-NMD isoform<br>(ENST00000383678.8)<br><br>NMD isoform(s) in SMG6/7 KD/KO<br>(ENST00000295864.9,<br>ENST00000488781.5,ENST00000485330.5)<br>PTC / PTC<br><br>Relevant isoform(s) in RNPS1 KD<br>(ENST00000383678.8, ENST00000485330.5)<br>PTC	✓	One of the GTPBP8 NMD sensitive isoforms is upregulated in RNPS1 KD<br>Reason: exon skipping

The isoform switch in GTPBP8 (control vs RNPS1_KD)

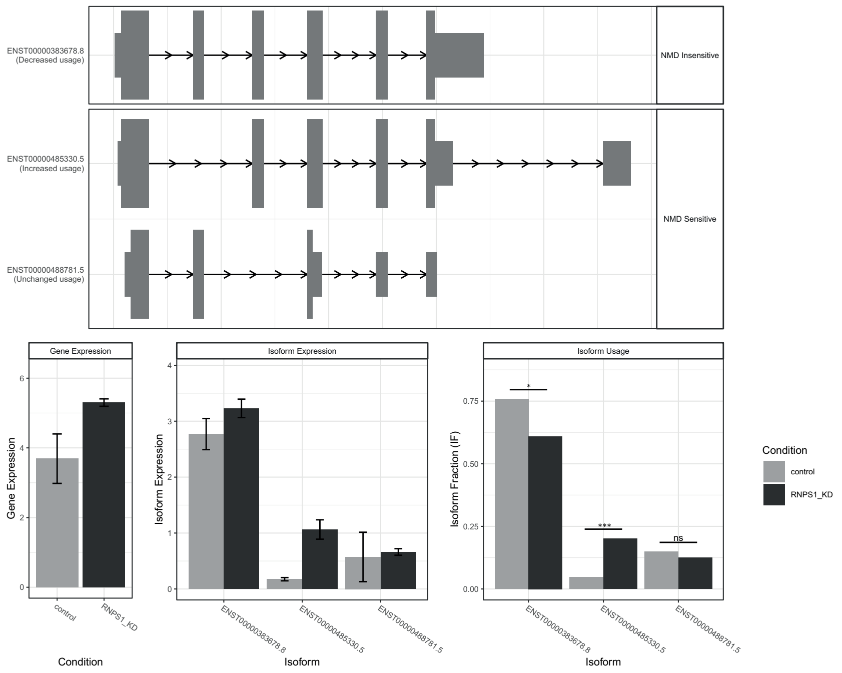

The isoform switch in GTPBP8 (control vs SMG7_KO_2_SMG6_KD)

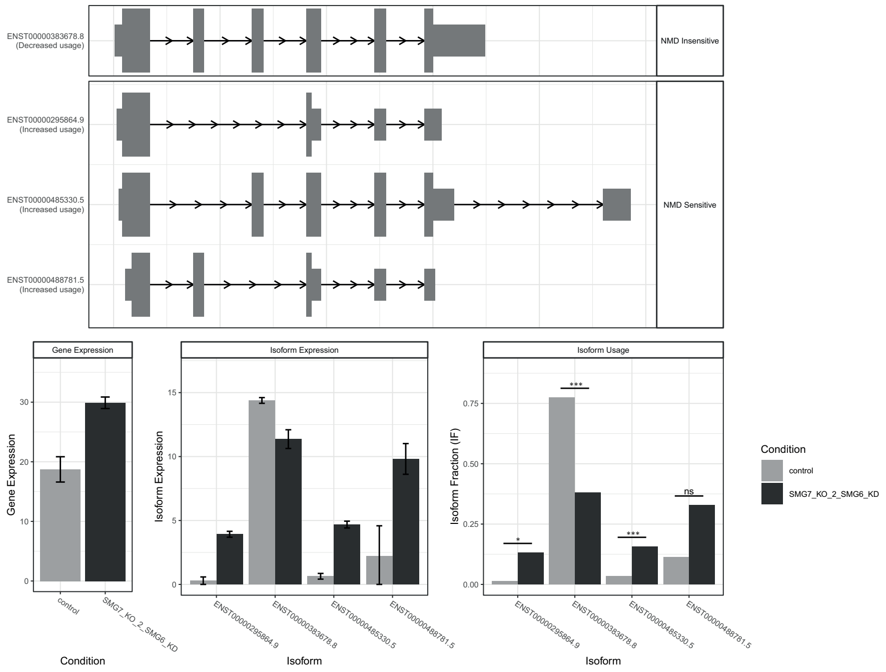

PCNP

Transcripts	RNPS1 KD - NMD relevant	Comment
Unspliced<br><br>Transcript isoforms:<br><br>Non-NMD isoform<br>(ENST00000265260.8)<br><br>NMD isoform(s) in SMG6/7 KD/KO<br>(ENST00000460231.5) PTC<br><br>Relevant isoform(s) in RNPS1 KD<br>(ENST00000265260.8, ENST00000460231.5) / PTC	✓	One of the PCNP NMD sensitive isoforms is upregulated in RNPS1 KD<br>Reason: alternative 3' SS

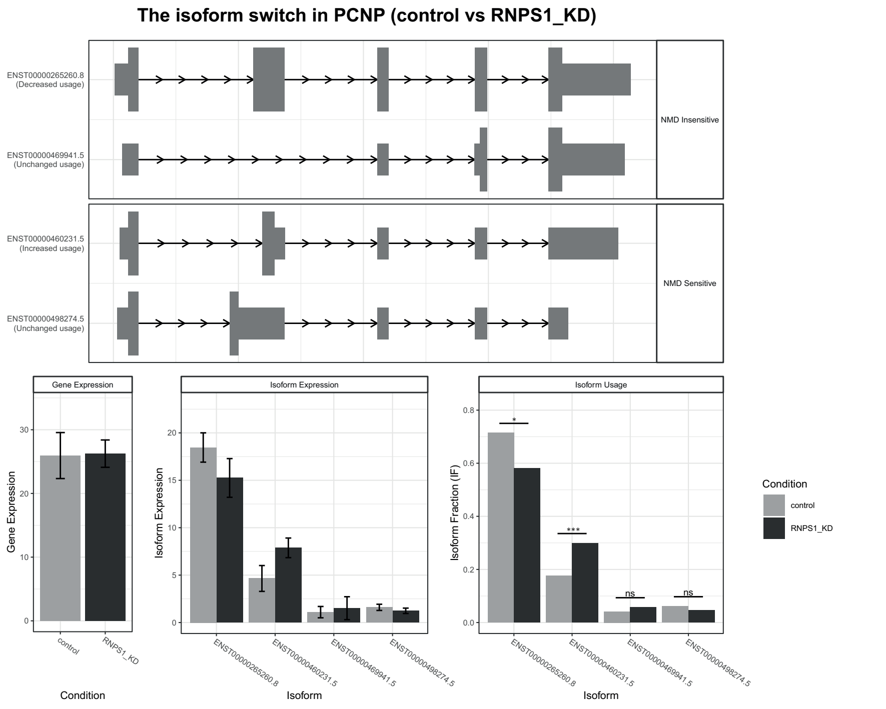

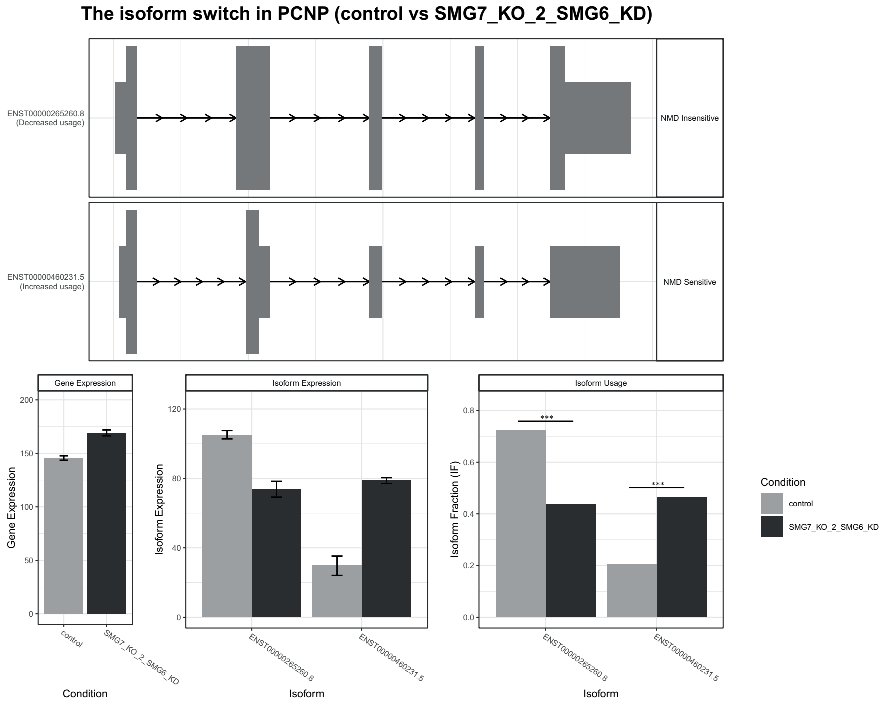

	Transcripts	RNPS1 KD - NMD relevant	Comment
**SNRNP48**	Unspliced<br><br>Transcript isoforms:<br>Non-NMD isoform<br>(ENST00000342415.6)<br><br>NMD isoform(s) in SMG6/7 KD/KO<br>(ENST00000634363.1) PTC<br><br>Relevant isoform(s) in RNPS1 KD<br>(ENST00000634363.1) PTC	✔	SNRNP48 NMD sensitive isoform is upregulated in RNPS1 KD<br>Reason: exon inclusion

The isoform switch in SNRNP48 (control vs RNPS1_KD)

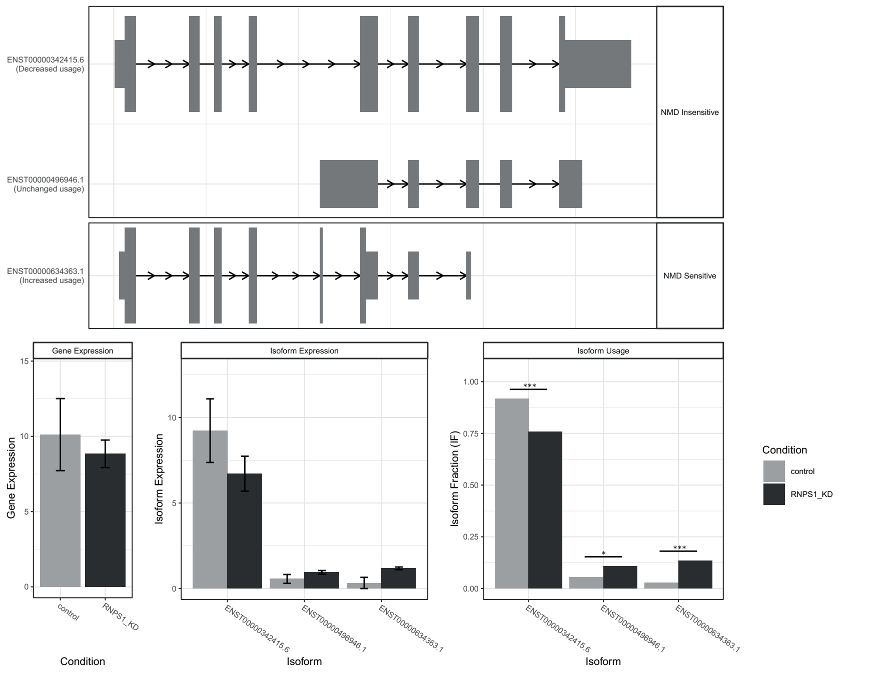

The isoform switch in SNRNP48 (control vs SMG7_KO_2_SMG6_KD)

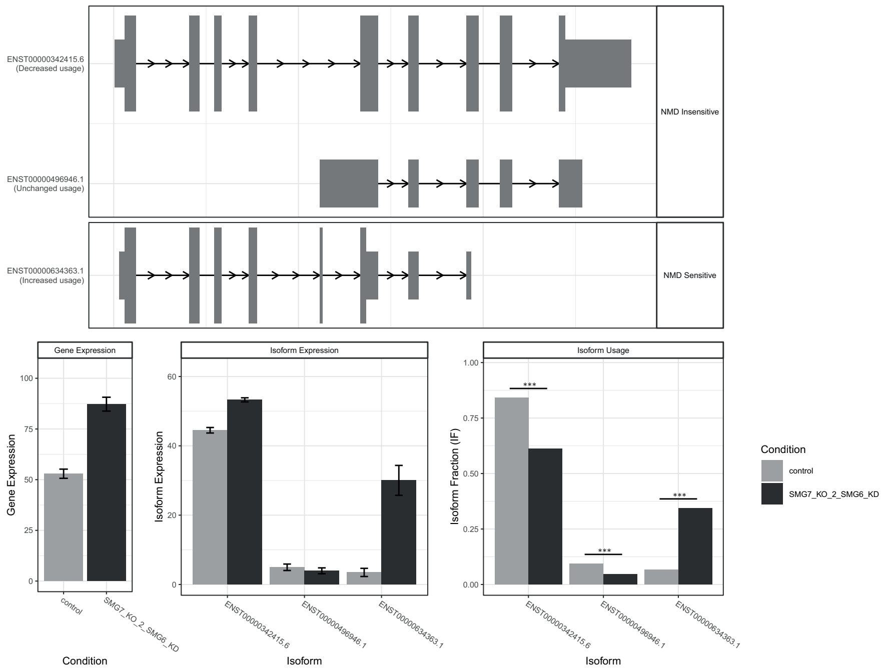

	Transcripts	RNPS1 KD - NMD relevant	Comment
**H2AX**	Not relevant	?	An isoform switch is mainly detected, since RNPS1 depletion leads to downregulation of the normal isoform. The NMD isoform is expressed in RNPS1 KD, but not upregulated compared to the control KD.

The isoform switch in H2AX (control vs RNPS1_KD)

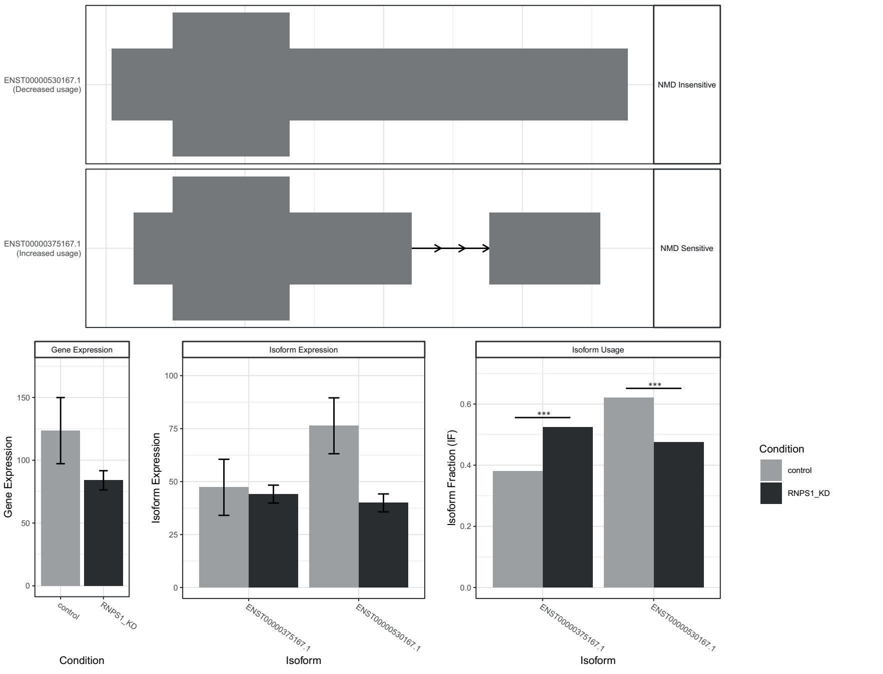

The isoform switch in H2AX (control vs SMG7_KO_2_SMG6_KD)

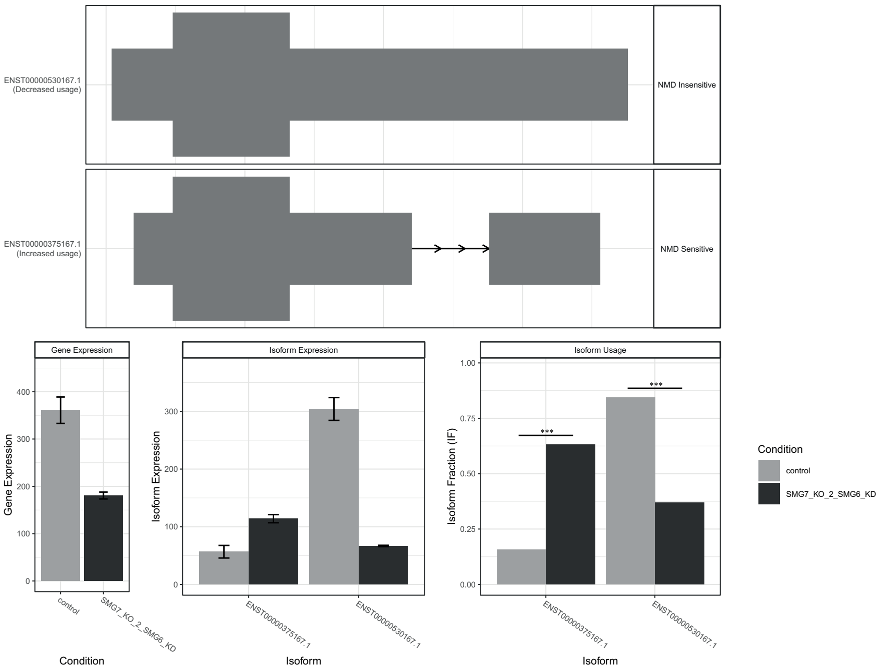

	Transcripts	RNPS1 KD - NMD relevant	Comment
**ASNS**	Not relevant	?	RNPS1 depletion leads to strong gene downregulation. NMD isoform is expressed in RNPS1 KD, but not upregulated compared to the control KD.

The isoform switch in ASNS (control vs RNPS1_KD)

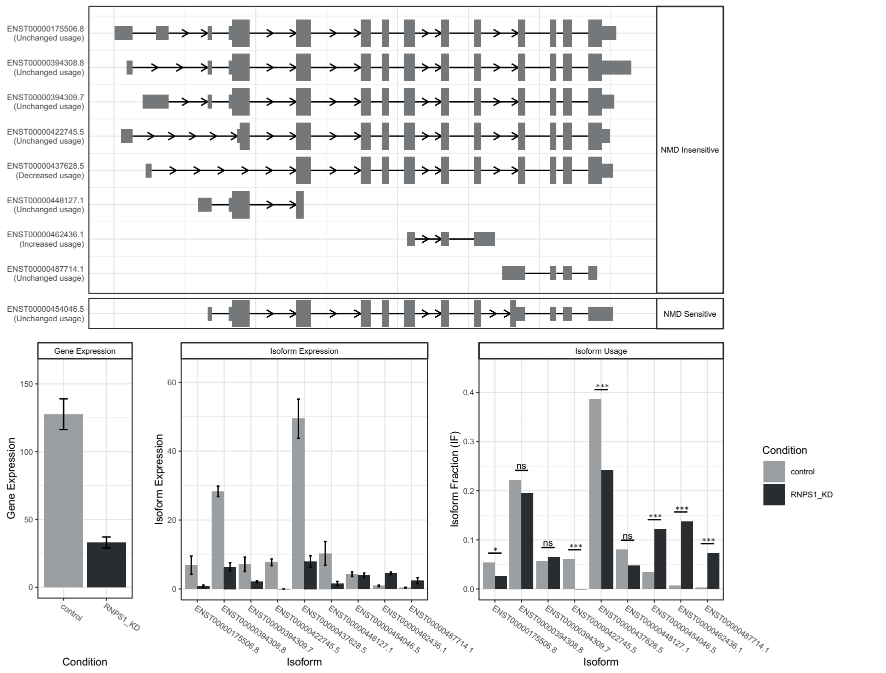

The isoform switch in ASNS (control vs SMG7_KO_2_SMG6_KD)

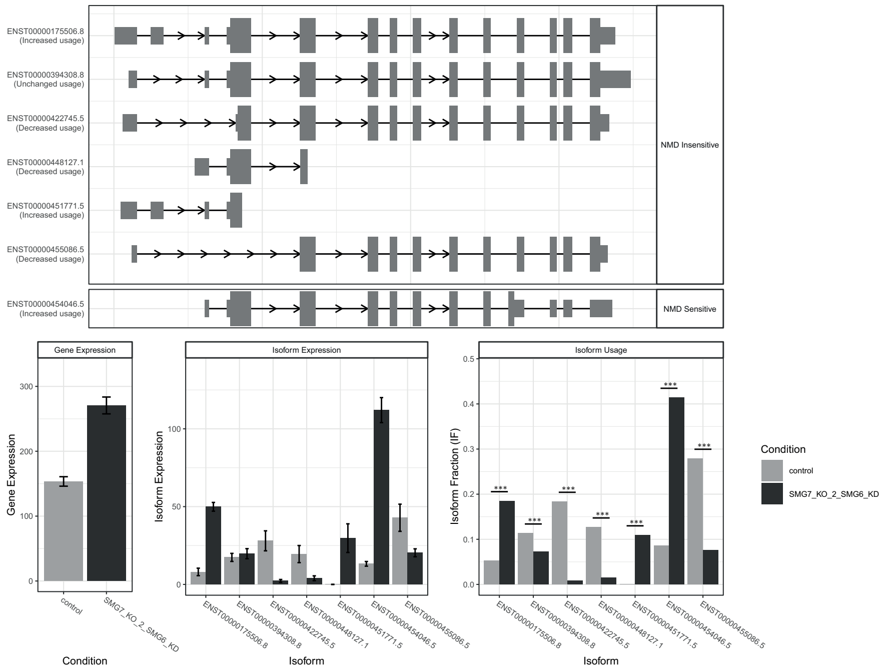

PXMP2	Transcripts	RNPS1 KD - NMD relevant	Comment
		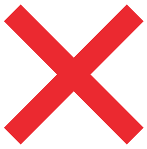	NMD sensitive isoform is downregulated in RNPS1 KD

The isoform switch in PXMP2 (control vs RNPS1_KD)

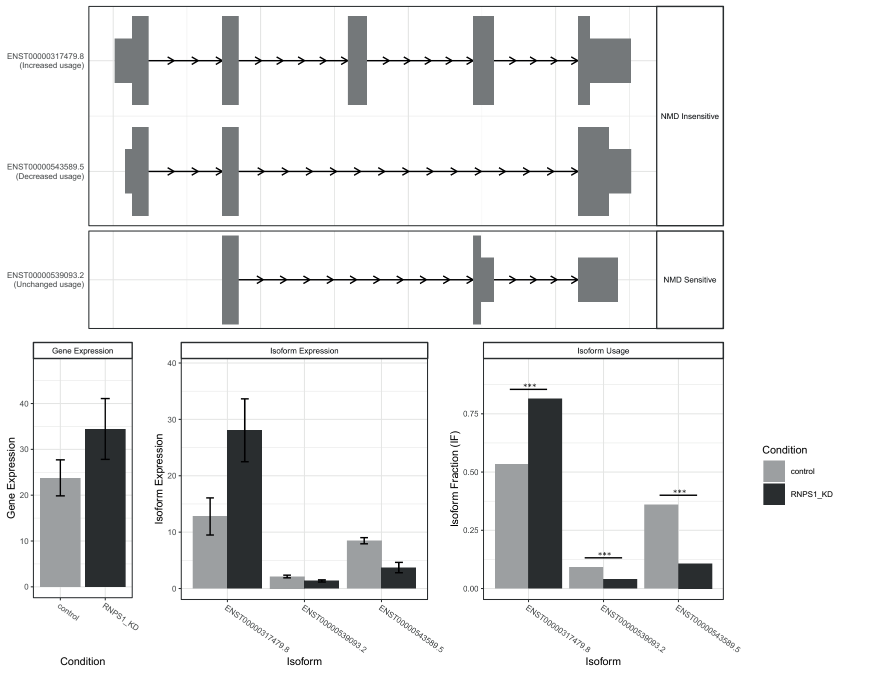

The isoform switch in PXMP2 (control vs SMG7_KO_2_SMG6_KD)

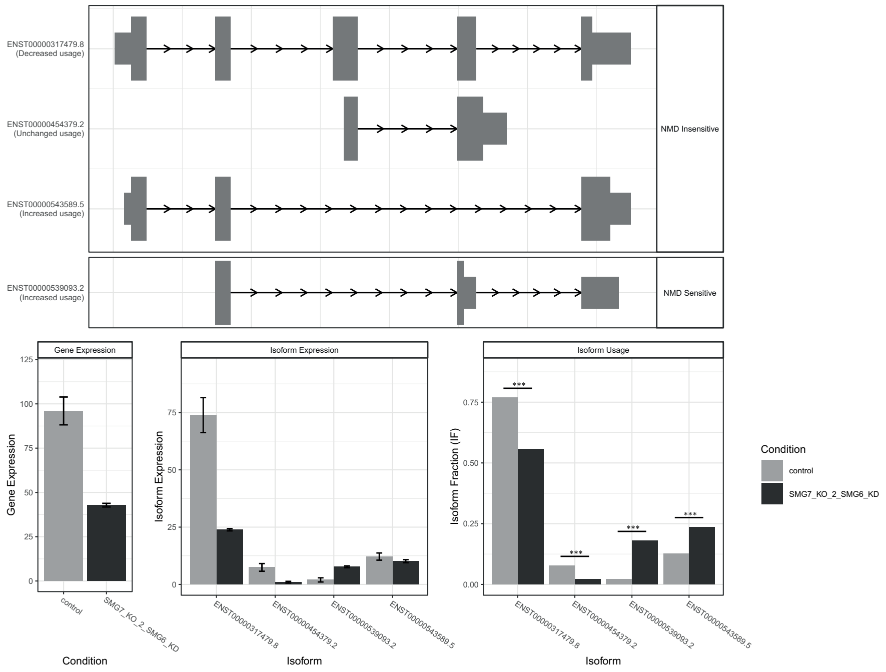

	Transcripts	RNPS1 KD - NMD relevant	Comment
**PTPMT1**		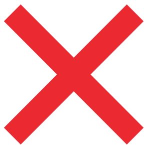	NMD sensitive isoform is downregulated in RNPS1 KD

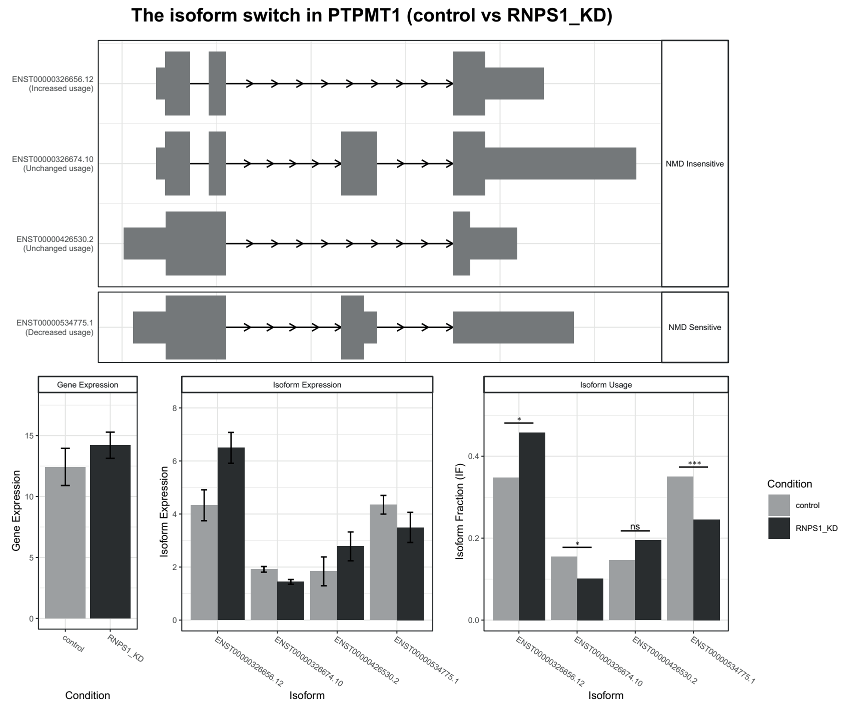

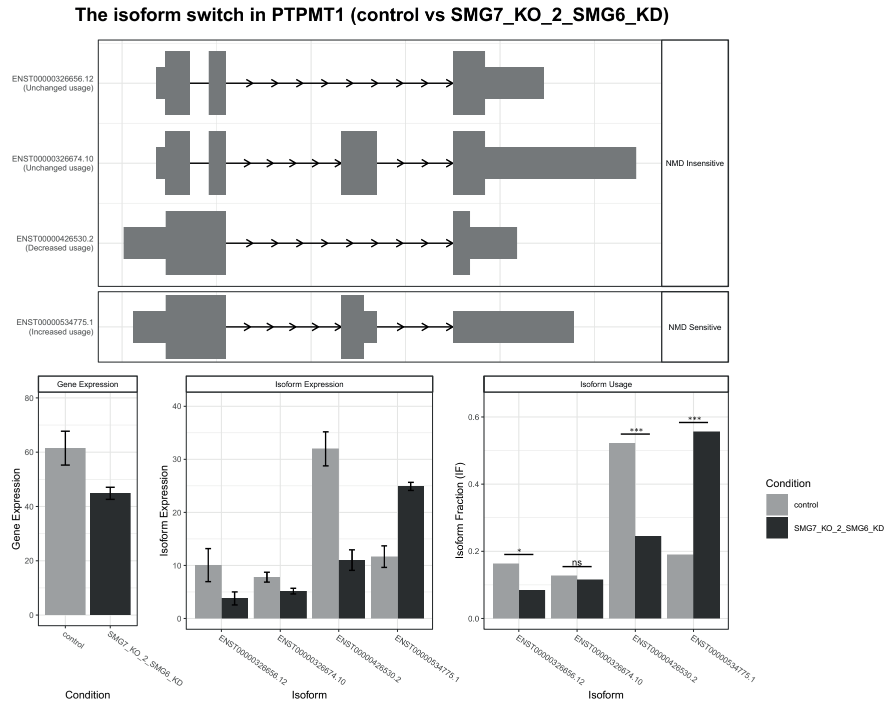

	Transcripts	RNPS1 KD - NMD relevant	Comment
**DNAJC5**	Not relevant	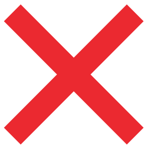	NMD sensitive isoform is downregulated in RNPS1 KD

The isoform switch in DNAJC5 (control vs RNPS1_KD)

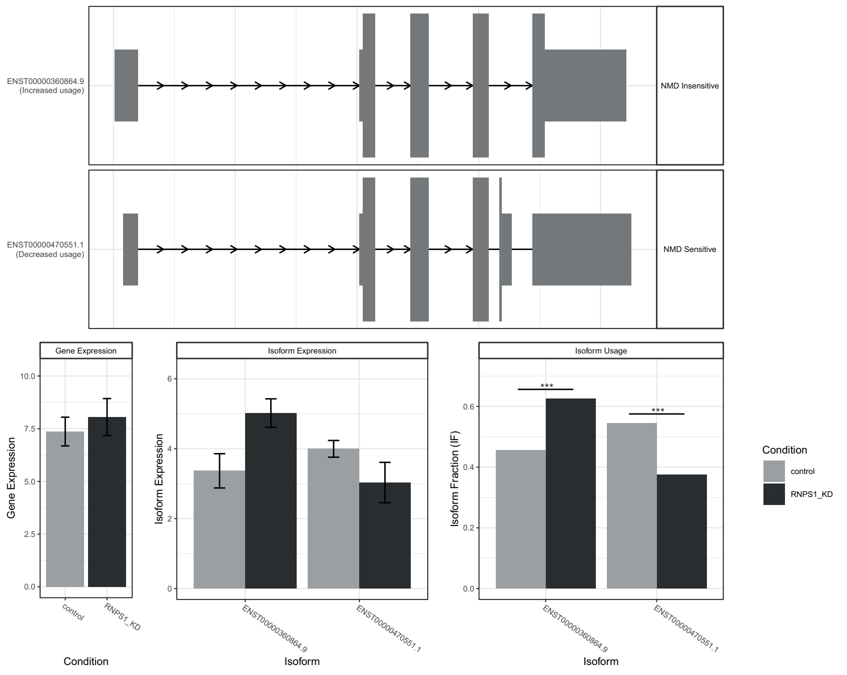

The isoform switch in DNAJC5 (control vs SMG7_KO_2_SMG6_KD)

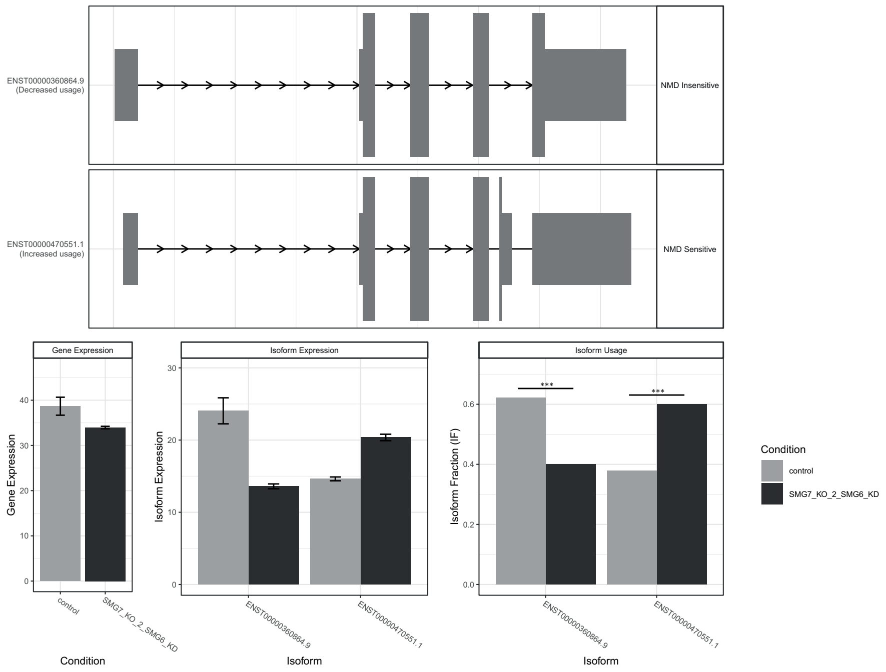

Transcripts	RNPS1 KD - NMD relevant	Comment

FAM234B

Unspliced

Transcript isoforms:

Non-NMD isoform
(ENST00000197268.13)

NMD isoform(s) in SMG6/7 KD/KO
(ENST00000416494.6)

Relevant isoform(s) in RNPS1 KD
(unannotated in GENCODE; no PTC)

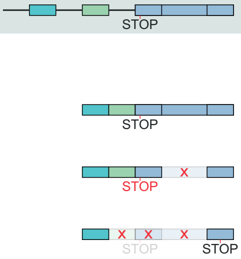

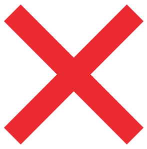

Unannotated skipping of two exons: erronous quantification of an NMD isoform that is not really upregulated in RNPS1 KD

The isoform switch in FAM234B (control vs RNPS1_KD)

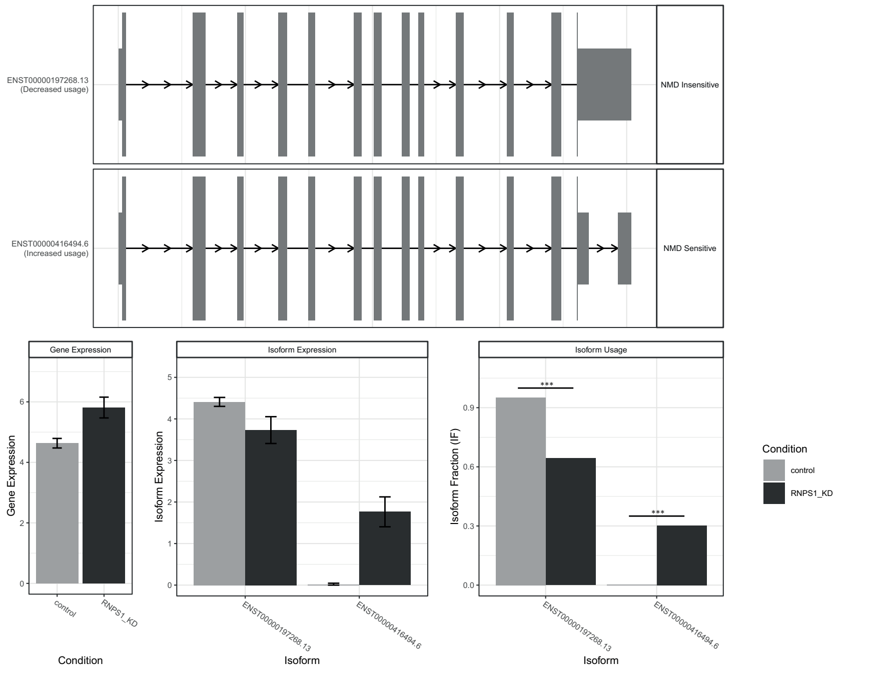

The isoform switch in FAM234B (control vs SMG7_KO_2_SMG6_KD)

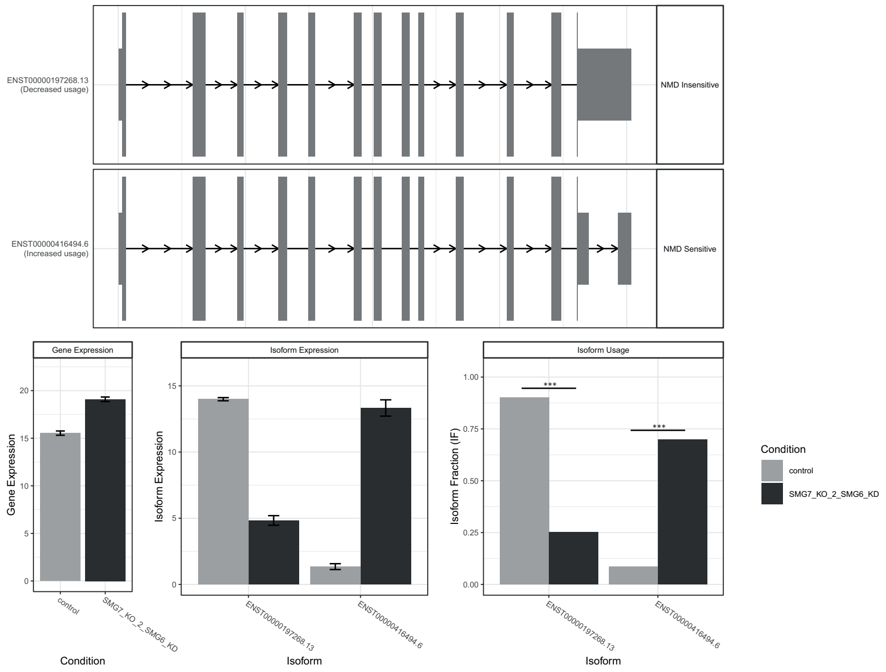

	Transcripts	RNPS1 KD - NMD relevant	Comment
**ATG5**	Unspliced<br><br>Transcript isoforms:<br>Non-NMD isoform (ENST00000369076.8)<br>NMD isoform(s) in SMG6/7 KD/KO (ENST00000646025.1) PTC<br>Relevant isoform(s) in RNPS1 KD (unannotated in GENCODE; not quantified)	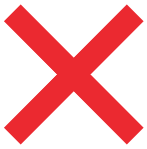	The annotated NMD-inducing isoform is not properly detected, potentially due to the unannotated inclusion of a novel small exon.

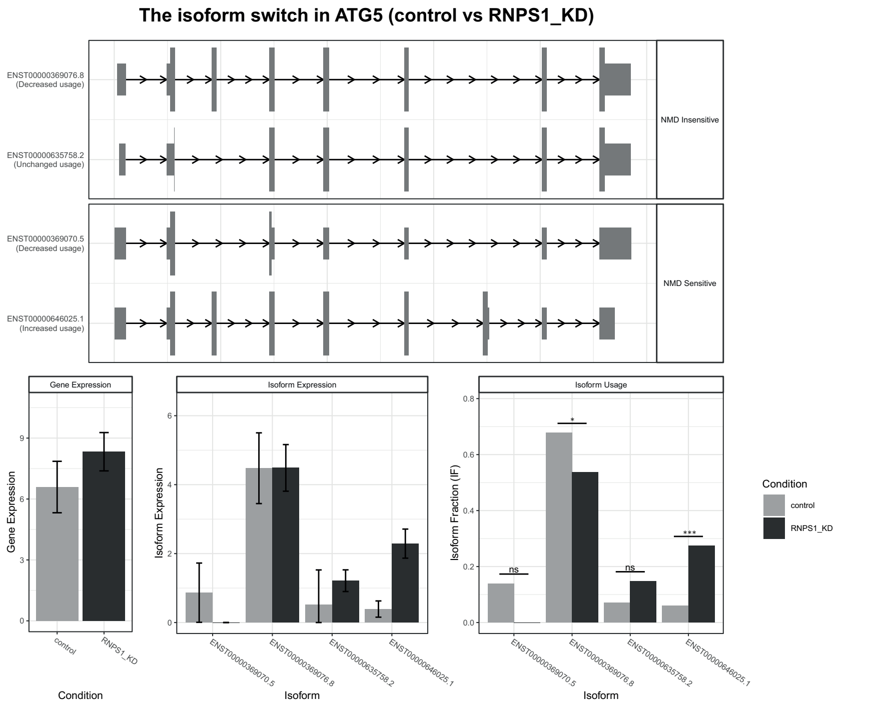

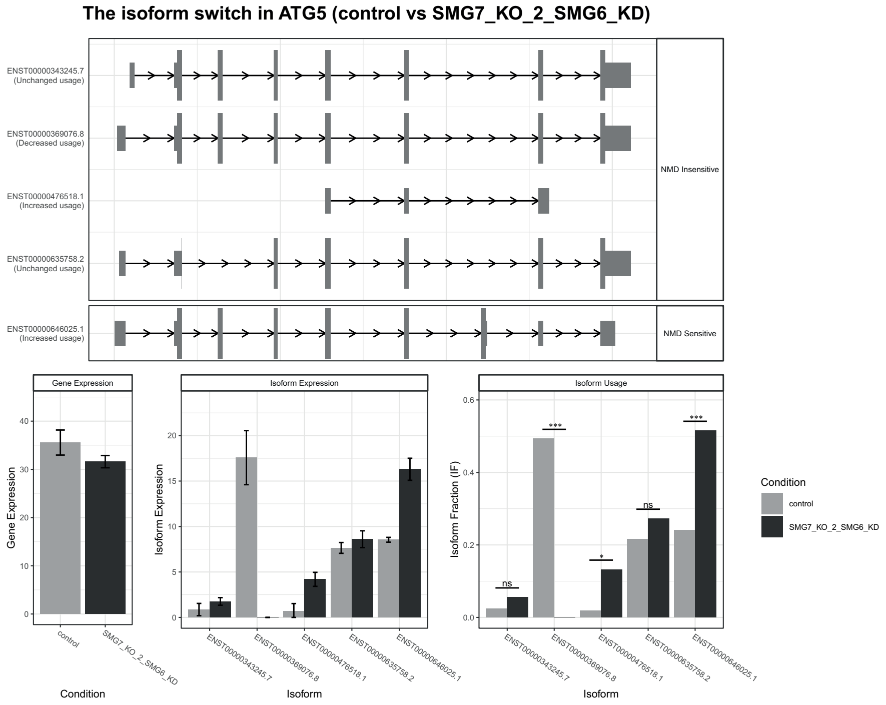

Transcripts	RNPS1 KD - NMD relevant	Comment

TMEM248

Unspliced

Transcript isoforms:

Non-NMD isoform
(ENST00000341567.8)

NMD isoform(s) in SMG6/7 KD/KO
(ENST00000433271.6, ENST00000341567.8)

Relevant isoform(s) in RNPS1 KD
(unannotated in GENCODE, ENST00000341567.8)

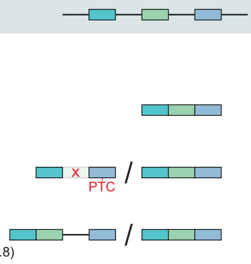

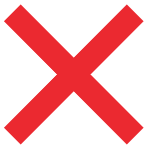

The NMD-inducing exon skipping event is not found in the RNPS1 KD, but rather an increase in intron retention.
We suspect problematic quantification/identification as the source for this misclassification

The isoform switch in TMEM248 (control vs RNPS1_KD)

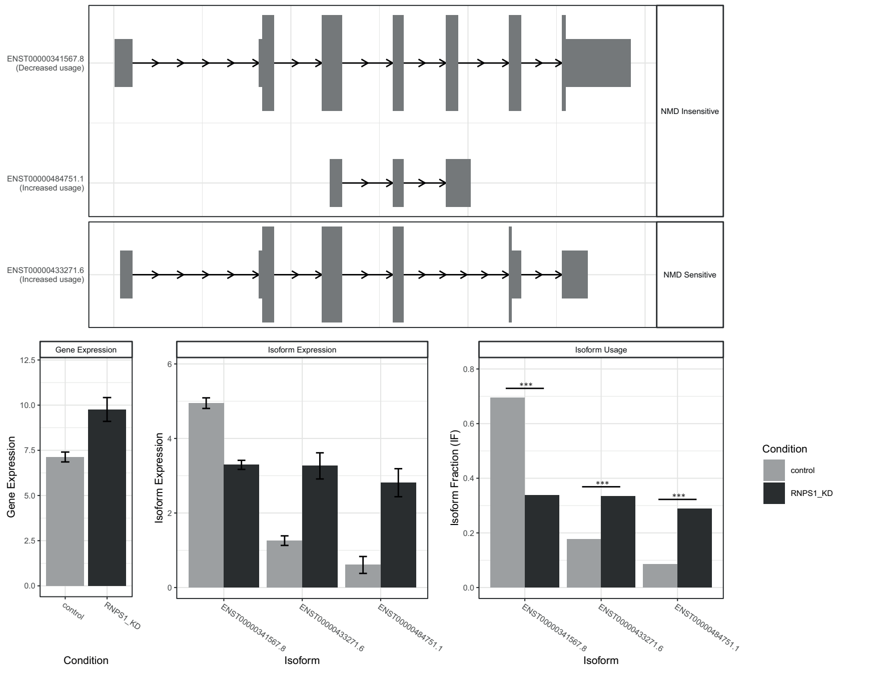

The isoform switch in TMEM248 (control vs SMG7_KO_2_SMG6_KD)

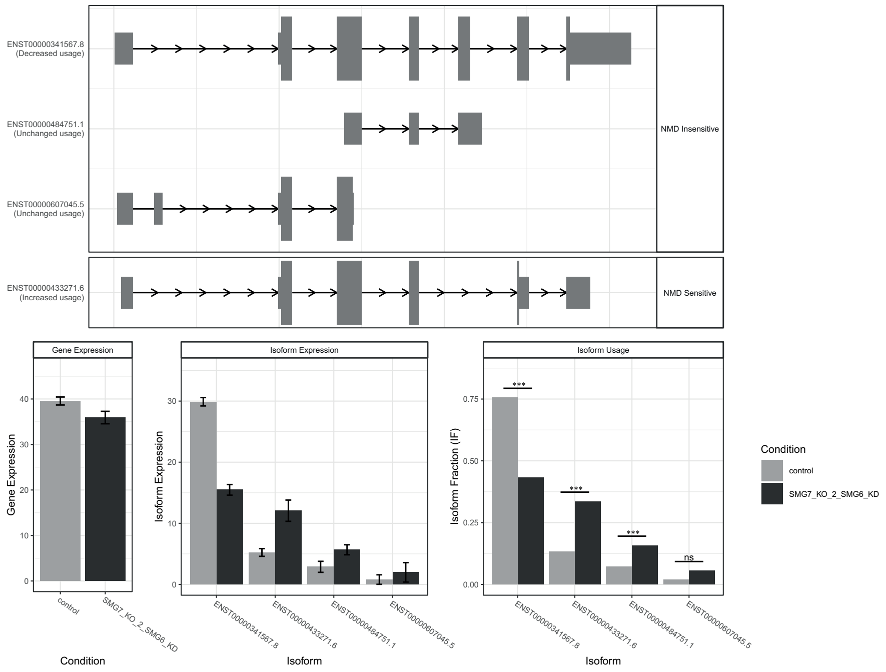

	Transcripts	RNPS1 KD - NMD relevant	Comment
**HSPA4**	Not relevant	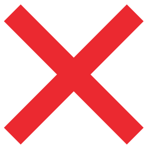	The annotated transcript was „retired“ by Ensembl. It contained small deletions in exons (leading to PTC), which were not found in the RNA-Seq data

The isoform switch in HSPA4 (control vs RNPS1_KD)

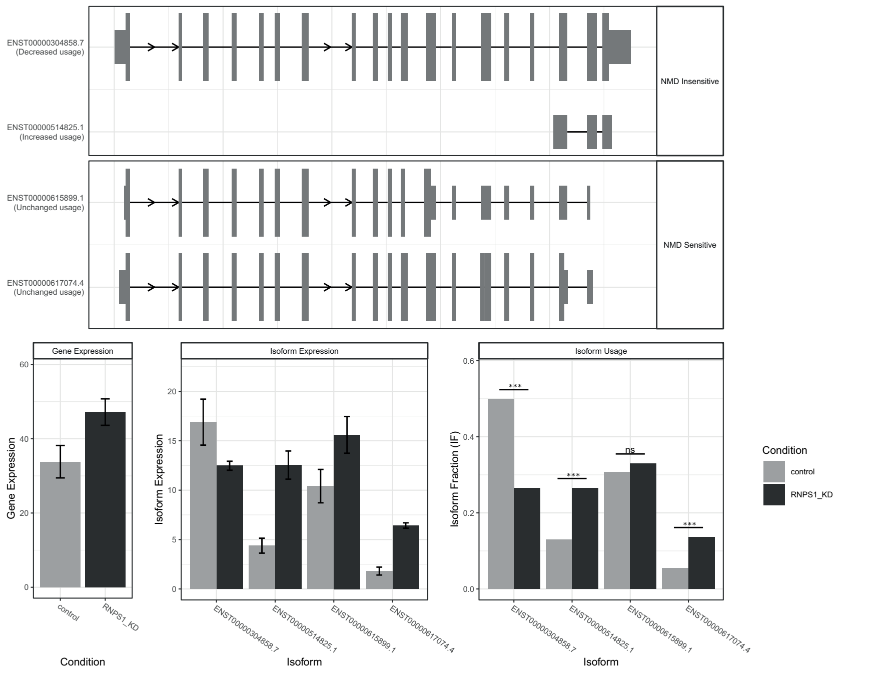

The isoform switch in HSPA4 (control vs SMG7_KO_2_SMG6_KD)

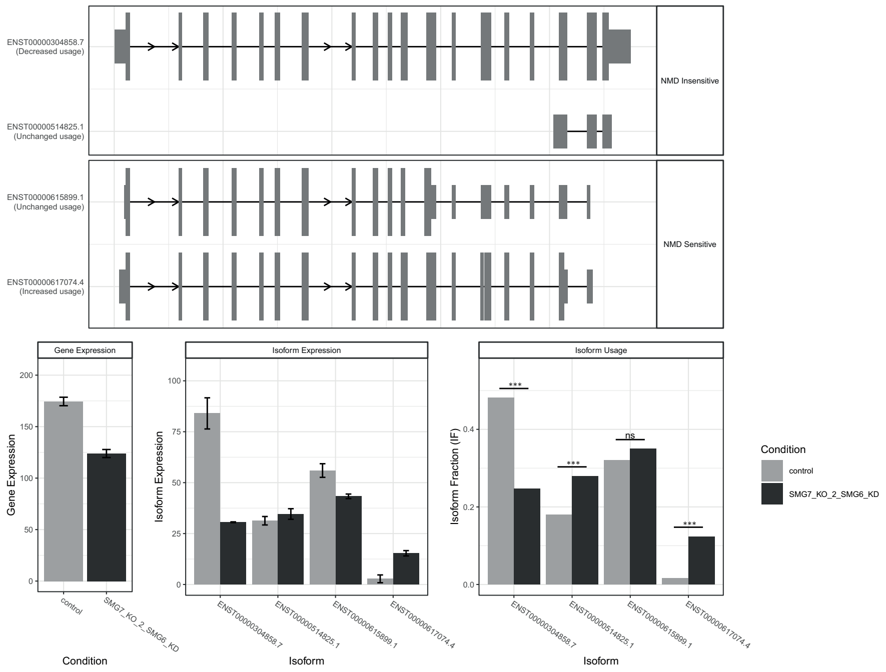

Supplement: gkac428_Supplemental_Files [file gkac428_supplemental_files.zip › Schlautmann_et_al_2022_Supplementary_Data.zip!/Schlautmann_et_al_2022_Supplementary_Dataset_1.pdf]
